# The Association of Gut Microbiota With *TRPM7* Genotype, Colorectal Polyps, and Magnesium

**DOI:** 10.1016/j.tjnut.2025.07.015

**Published:** 2025-07-31

**Authors:** Shan Sun, Xiangzhu Zhu, Xiang Huang, Chang Yu, Timothy Su, Harvey J Murff, Reid M Ness, M. Andrea Azcarate-Peril, Martha J Shrubsole, Qi Dai

**Affiliations:** 1 Department of Bioinformatics and Genomics, University of North Carolina at Charlotte, Charlotte, North Carolina, United States; 2 Division of Epidemiology, Department of Medicine, Vanderbilt University Medical Center, Nashville, Tennessee, United States; 3 Division of Biostatistics, Department of Population Health, NYU Grossman School of Medicine, New York, New York, United States; 4 Division of Geriatric Medicine, Department of Medicine, Vanderbilt University Medical Center, Nashville, Tennessee, United States; 5 Division of Gastroenterology, Hepatology and Nutrition, Department of Medicine, Vanderbilt University Medical Center, Nashville, Tennessee, United States; 6 Department of Medicine and Microbiome Core, School of Medicine, University of North Carolina at Chapel Hill, Chapel Hill, North Carolina, United States

**Keywords:** gut microbiota, *TRPM7*, colorectal polyps, magnesium, precision-based randomized trial

## Abstract

**Background:**

We previously reported that individuals with the transient receptor potential melastatin 7 (*TRPM7*) GA/AA genotype and consumed diets high in Ca:Mg ratio had increased risk of colorectal polyps.

**Objectives:**

The aim was to identify whether the gut microbiota plays a role in the association of *TRPM7* genotype, Ca:Mg intake ratio, and risk of colorectal polyps.

**Methods:**

We analyzed the gut microbiota of 240 participants in a double-blind 2 × 2 factorial (*TRPM7* genotype and Ca:Mg intake ratios) randomized trial by sequencing the stool, rectal swab, and rectal mucosa tissue samples of each participant.

**Results:**

The gut microbiota of participants with the GA genotype significantly differed from those with the GG genotype in all 3 sample types, with an altered abundance of *Prevotella* and *Bacteroides* in swab samples. *Prevotella* in rectal mucosa and *Bacteroides* in swab were associated with an increased risk of metachronous colorectal polyps. Optimizing high-diet Ca:Mg ratios to 2.3 through Mg supplementation resulted in a reduced abundance of *Prevotella* in rectal swabs and *Bacteroides* in stool samples. We identified multiple microbes in all 3 sample types linked to risk of metachronous colorectal polyps.

**Conclusions:**

Our findings indicate that the gut microbiota in stool, rectal swab, and mucosa are associated with risk of metachronous colorectal polyps, and diet changes could modify the abundance of *TRPM7-*related microbes.

This study was registered at clinicaltrials.gov as NCT01105169 (https://clinicaltrials.gov/study/NCT01105169).

## Introduction

Transient receptor potential melastatin (TRPM)7, a magnesium-regulated chanzyme [1] possessing both ion channel and kinase activities [2], has a much stronger affinity to Mg^2+^ than that to Ca^2+^ [[3], [4], [5]]. We previously reported that the Thr1482Ile functional variant (or G→A) [6] in the *TRPM7* was associated with significantly increased risks of both colorectal adenomas and serrated polyps in those with high Ca:Mg intake ratios [7]. The *TRPM7* gene was also observed to possess “driver” mutations that contribute to the development of multiple cancers [8]. In recent years, TRPM7 was identified as a downstream effector of mixed lineage kinase like (MLKL)-mediated necroptosis [9], a new form of programmed cell deaths [10]. In contrast to canonical programmed cell death (i.e., apoptosis) [10], MLKL-mediated necroptosis elicits robust adaptive immune responses [11,12], including the inflammatory cascade [10] against bacterial/viral infection [13]. Substantial evidence has linked inflammation in the colorectum to colorectal carcinogenesis [14,15]. In a recent report, expression levels of cyclooxygenase (COX)-2, particularly COX-2 combined with TRPM7 and MLKL, in rectal mucosa were longitudinally associated with increased risks of metachronous adenomas and serrated polyps [16].

Gut microbiota plays a crucial role in human health and diseases. It interacts with host immune system and contributes to the transition between inflammatory and noninflammatory states of gut. A dysbiotic microbial community can weaken the intestinal barrier and trigger inflammation [17]. Mucin glycans protect the gut epithelium from potential pathogens [18,19] and regulate the composition of the mucin-degrading microbiota [[20], [21], [22]]. Sulfation provides enhanced protection to mucin glycans from mucin-degrading bacteria without desulfating capacities [18,23] and serves as a mucosal barrier against colorectal inflammation [24]. Overactive mucin degradation activity of gut microbiota can contribute to a thinner and more permeable colonic mucus barrier and increase susceptibility to pathogen infection and inflammation [25]. Microbial desulfating enzymes are highly conserved and are activated by higher Ca:Mg ratios [[26], [27], [28]]. Thus, we hypothesize that high Ca:Mg intake ratios can lead to overgrowth of desulfating microbes in the colorectum and colorectal carcinogenesis, particularly in those with the Thr1482Ile variant of the *TRPM7* gene, because the Thr1482Ile variant caused reduced TRPM7 activity [6], whereas TRPM7-deficient cells led to Mg deficiency.

To investigate the potential roles of human gut microbiota in the association of *TRPM7* genotype, high Ca:Mg intake ratios, and colorectal adenomas and serrated polyps, we performed whole genome sequencing on stool, rectal swab, and mucosal tissue samples of participants in the Personalized Prevention of Colorectal Cancer Trial (PPCCT) designed to examine the interaction between Ca:Mg intake ratios and the *TRPM7* genotype in affecting biomarkers for colorectal carcinogenesis. First, we investigated whether optimizing Ca:Mg intake ratios to 2.3 through Mg supplement modified biomarkers related to colorectal carcinogenesis (e.g., COX-2, TRPM7, and MLKL) in rectal mucosa in a precision-based randomized trial randomly assigned by *TRPM7* genotype and Mg treatment. Next, we identified the *TRPM7* genotype–associated microbes in a study specifically conducted in participants with Ca:Mg intake ratios of ≥2.6. Among the 227 participants with a history of polyp, adenoma, and serrated polyp diagnosed before the baseline, 124 participants had 1 or more colonoscopy after they completed the trial and were included in the metachronous polyp analysis. Participants without a polyp during the surveillance colonoscopy completed posttrial were defined as metachronous-free controls, and participants with a diagnosis of polyp during the surveillance colonoscopy posttrial were classified as metachronous polyp cases. We examined whether the microbes that were significantly associated with the genotype were associated with risk of metachronous adenoma/serrated polyps. We also evaluated whether optimizing the Ca:Mg intake ratio to 2.3 through Mg supplementation altered the abundance of 2 desulfating microbes that were associated with *TRPM7* genotype and risk of metachronous polyps. We also performed an analysis to analyze which other taxa linked to risk of metachronous polyps independent of *TRPM7* genotype.

## Methods

### Precision-based randomized trial

This is an ancillary study of the parent study, the PPCCT (NCT01105169 at clinicatTrials.gov). The PPCCT is a double-blind 2 × 2 factorial randomized controlled trial conducted at Vanderbilt University Medical Center (VUMC), Nashville, Tennessee, designed to examine the interaction between Ca:Mg intake ratios and the Thr1482Ile functional variant in the *TRPM7* gene [7] in affecting biomarkers for colorectal carcinogenesis. The study was approved by the Vanderbilt institutional review board (No.: 100106), and informed consent was obtained from all individual participants. The detailed design has been reported elsewhere [29,30]. In brief, participants aged 40–85 y were recruited from the VUMC patient sources including the following: *1*) 236 individuals with adenomas or serrated polyps (i.e., hyperplastic polyps) diagnosed from 1998 to 2014 and *2*) 14 polyp-free individuals with a high risk of colorectal cancer. Two 24-h dietary recalls, which recorded all foods and beverages and their amounts over the 24-h period, were performed for all participants at study baseline to calculate intake levels of Ca, Mg, and the Ca:Mg intake ratios for each recall. The means were calculated for the Ca, Mg, and Ca:Mg ratio intakes across the 2 recalls. Eligible participants had a Ca intake of ≥700 and <2000 mg/d and in whom the Ca:Mg intake ratio was ≥2.6.

Eligible participants were assigned to Mg treatment or placebo according to the randomization schedule. The randomization procedure used randomly assigned blocks of 2 or 4 to allocate subjects in a 1:1 ratio to 2 treatment arms—magnesium treatment or placebo—within 3 strata defined by the *TRPM7* genotype: GG, GA, and AA. Eligible individuals were enrolled sequentially and were assigned sequentially to receive magnesium treatment or placebo according to the randomization schedule. The participants assigned to magnesium treatment received a personalized dose of Mg supplementation that would reduce the Ca:Mg intake ratio to ∼2.3. This ratio was selected because previous studies suggested that intakes of both calcium and magnesium were beneficial when Ca:Mg intake ratios were between 1.7 and 2.6–2.8 [7,29,[31], [32], [33], [34], [35], [36]]. Three sizes of Mg glycinate capsules were filled by the Vanderbilt Investigational Pharmacy personnel following USP 797 conditions according to the compounding instructions. Identical-appearing placebos (i.e., microcrystalline cellulose) were made to match Mg capsules. Participants, study investigators, and staff were blinded to the assigned interventions. The intervention period was 12 wk. Anthropometric measurements (weight, height, and waist and hip circumference) were measured at least twice at each study visit. The proposed primary end points for the PPCCT were biomarkers related to colorectal carcinogenesis in rectal mucosa, including necroptosis biomarkers (i.e., TRPM7 and phosphorylated mixed lineage kinase like [pMLKL]), inflammation biomarkers (i.e., COX-2), apoptosis biomarkers (i.e., BCL2-associated X [BAX] and terminal deoxynucleotidyl transferase dUTP nick end labeling [TUNEL]) and cell proliferation biomarkers (i.e., antigen Kiel 67 [Ki67]), as well as the ratios of Ki67:BAX and the ratio of Ki67:TUNEL in rectal mucosa.

Of the 250 randomly assigned participants that started treatments, 239 completed the trial, with 11 participants finishing part of the study before withdrawing [29]. Six of the withdrawals were due to self-reported adverse events (4 withdrawals in the treatment arm and 2 in the placebo arm). One of them had donated biospecimens at baseline and at the end of the trial. Therefore, 240 participants were included who had biospecimens collected at baseline and at the end of the trial. Compliance with the pill regimen was very high for both the placebo and treatment arms [mean (SD)] based on pill counts were 97.3% (4.4) and 97.5% (3.9), respectively; *P* = 0.83 for difference between the arms).

### Genotyping assay for the *TRPM7* gene

The detailed approach was previously reported [10]. In brief, DNA was extracted from buffy coat fractions or cheek cells by using a QIAamp DNA mini-kit (Qiagen) according to the manufacturer’s protocol. The allelic discrimination of the rs8042919 polymorphism (i.e., Thr1482Ile functional polymorphism [G→A] genotyping) in the *TRPM7* gene was assessed by using the TaqMan genotyping assay (Assay ID: C_25756319_10; Applied Biosystems). Baseline characteristics for continuous variables (mean ± SD, median, and IQR) and categorical variables (count and percentages) were reported for *TRPM7* genotype. Generalized linear models and Pearson correlation were used for continuous variables and Kruskal–Wallis and χ^2^ tests were conducted to compare categorical variables between genotype groups.

#### Assays for immunohistochemistry biomarkers in rectal biopsies

During the study visits at baseline and the end of the PPCCT, biopsy tissues from the quadrants in the mid-rectum were collected and put in 10% neutral buffered formalin immediately for 24 h, followed by standard paraffin embedding. Immunohistochemical (IHC) staining was conducted following DAKO EnVision+ System-HRP protocol (Rabbit/Mouse kit, Code K4010/Code K4007) to detect TRPM7, pMLKL, BAX, COX-2, and Ki67. Detailed antibody information and validation data were included in Supplemental Table 1. Positive control tissues included human skin (for pMLKL), liver (for BAX), and a laboratory-constructed tissue microarray with normal and colorectal cancer (CRC) tissues for TRPM7, COX-2, and Ki67. For negative controls, phosphate-buffered saline was used to replace primary antibodies. Both positive and negative control slides were processed concurrently with each staining batch. Apoptosis was detected using the Promega DeadEnd Colorimetric TUNEL Assay (Promega BioScience; Cat# G7130). Biomarker expression was quantified separately in the upper (luminal-facing, 40%) and lower (submucosa-adjacent, 60%) zones of the rectal crypts using a computer-aided imaging analysis system comprising an Olympus BX40 microscope and BioQuant NOVA Prime imaging software (BioQuant). Detailed methodologies for antibody validation, TUNEL assay modification, and zonal quantitative imaging analysis have been published elsewhere [16].

#### Whole genome shotgun sequencing of stool, swab and rectal biopsies

We collected stool, rectal swab, and mucosal tissue samples through rectal biopsy for the 240 participants at home or in-person study visits at the beginning of the trial (baseline) and at the end of the trial. Whole genome shotgun sequencing was performed on the stool, rectal swab, and mucosal tissue samples collected at both the beginning and the end of the trial as previously described [37,38]. The details of sequencing methods are described in Supplemental Methods. The metagenome sequencing files of stool, rectal swab, and mucosal tissue samples at both the beginning and the end of the trial were processed with KneadData for quality control and removal of human DNA contamination. MetaPhlAn2 was used for analyzing the taxonomic profiles of shotgun metagenome sequences [39], and HUMAnN2 was used for functional profiling including the abundance of functional pathways [40]. The sequences generated in this study can be accessed at National Center for Biotechnology Information (NCBI) BioProject PRJNA693850.

### Statistical analyses of *TRPM7* genotype and magnesium treatment

The primary outcome is the changes of gut microbiota, measured at the community level as β-diversity and at individual taxa level as the relative abundance of each taxon. For each of the 3 sample types (stool, rectal swab, and rectal mucosa), Bray-Curtis dissimilarity between samples was calculated based on the genus abundance, stratified and unstratified pathway abundance, and visualized with principal coordinate analysis (PCoA). Permutational multivariate ANOVA (PERMANOVA) of gut taxonomic and functional profiles was used to test their associations with *TRPM7* genotypes. The genus abundance matrix was used as the dependent variable in the taxonomic model, and the functional pathway abundance matrix was used as the dependent variable in the functional model. Because of the insufficient statistical power due to sample size, we combined the pretreatment and posttreatment samples for this analysis. To verify this finding, we also analyzed the pretreatment and posttreatment samples separately with the PERMANOVA test. We also visualized the separations between GG and GA genotypes in the pretreatment and posttreatment samples with PCoA.

In addition, we also tested the association between each taxa/pathway and genotypes with univariate nonparametric Wilcoxon tests, where the abundance of individual taxa/pathway was specified as the dependent variable. *P* values were adjusted with the Benjamini–Hochberg method to correct for multiple comparisons. Because of the limited statistical power with 240 participants, we pooled the pretrial and posttrial time points for the genotype-related analyses. Because the BMI (in kg/m^2^) of participants with GG and GA genotypes were different, we also ran a set of linear regression models with adjustment for BMI, where the abundance of individual taxa was used as dependent variable. Linear regression models of gut microbiota and biomarkers in rectal mucosa were used to test their association with assignment arm (Mg supplement compared with placebo) to examine the effect of reducing Ca:Mg intake ratios to 2.3. This model was further stratified by *TRPM7* genotype, and the models were repeated after adjustment for age, sex, and baseline levels. In these analyses, the abundance of individual taxa or biomarkers was used as the dependent variable. All *P* values are 2 sided, and statistical significance was determined using an α level of 0.05. The data analyses were performed with software SAS Enterprise Guide 7.1 (version 9.4; SAS Institute).

### Metachronous colorectal polyp/adenoma

**FIGURE 1:**
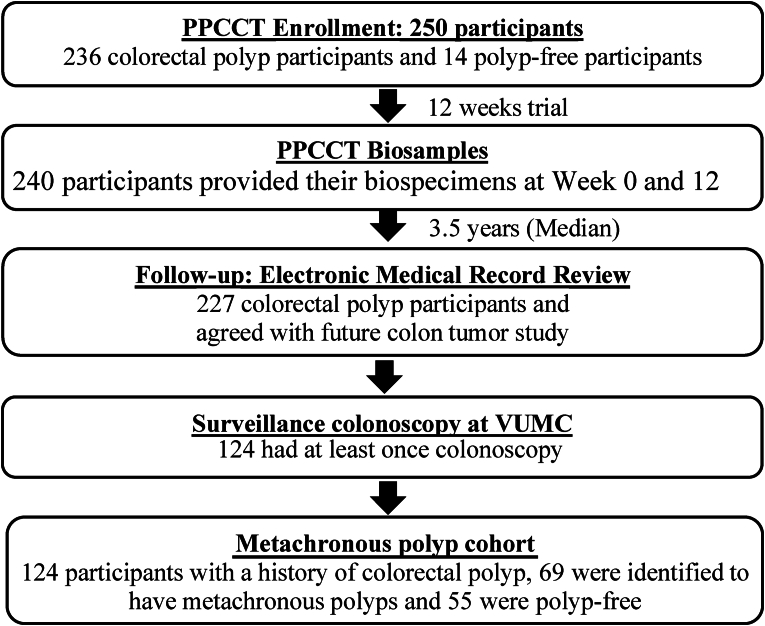
Study population flow diagram (CONSORT). PPCCT, Personalized Prevention of Colorectal Cancer Trial; VUMC, Vanderbilt University Medical Center.

The median follow-up time was 3.5 y after the completion of the PPCCT. Metachronous adenoma cases had a history of adenoma and a subsequent metachronous adenoma. Metachronous serrated polyp cases had a history of serrated polyp and a subsequent metachronous serrated polyp. A flow chart of the study population in the PPCCT is shown in Figure 1. During 2017–2018, we completed an extensive review of electronic medical records at VUMC to gather colonoscopy and pathology reports for the 240 PPCCT participants who completed the trial and had biospecimens collected at the baseline and the end of the trial. The colonoscopy and pathology reports provided detailed information on the procedure and on number, size, type, histology, degree of atypia, and location of polyps. In this analysis, 227 participants with a history of polyp, adenoma, and serrated polyp diagnosed before the baseline of the PPCCT were defined as participants with *1*) adenoma and/or serrated polyp; *2*) tubular or tubulovillous or villous adenoma; and *3*) serrated polyp (hyperplastic polyp or sessile serrated lesion), respectively. Further, 124 participants had 1 or more colonoscopy at VUMC after they completed the PPCCT and were included in the metachronous polyp analysis. Participants without a polyp during the surveillance colonoscopy completed post-PPCCT were defined as metachronous-free controls for this analysis, and participants with a diagnosis of polyp during the surveillance colonoscopy post-PPCCT were classified as metachronous polyp cases.

### Statistical analyses of microbial association with metachronous colorectal polyp/adenoma

The secondary outcome in this study is the colorectal polyps. Unconditional logistic regression models of metachronous colorectal adenoma/serrated polyps were used to estimate odds ratios and their 95% CI as a measure of the strength of the associations with the abundance of *TRPM7*-associated microbiota. Abundance of microbiota were categorized into tertiles based on the distribution of the controls in all analyses. The first model was adjusted only for age. The second model was additionally adjusted for other potentially confounding factors, including sex and BMI. Tests for trend across tertiles were performed in logistic regression models by assigning the score *j* to the *j*th level of the variable selected. Stratified analyses by the *TRPM7* genotype were conducted. Formal multiplicative interactions were also evaluated in logistic regression models by likelihood ratio tests. For an untargeted analysis, we conducted analyses to test the associations of metachronous colorectal adenoma/serrated polyps with the abundance of all genera and species. As not all microbial abundance met the assumption of normal distribution, we used nonparametric Wilcoxon tests and the continuous abundance of taxa with taxa abundance as the dependent variable. *P* values were corrected for multiple hypotheses testing with the Benjamini–Hochberg method.

## Results

### Baseline demographic characteristics by *TRPM7* genotype

We found that the demographic characteristics and other factors, including sex, age, educational achievement, income, use of antibiotics and nonsteroidal anti-inflammatory drugs and intakes of total energy, were not significantly different by *TRPM7* Thr1482Ile genotype (Supplemental Table 2). However, we found participants with the GA genotype had significantly higher BMI (31.4) than those with the GG genotype (29.6).

### Effects of Mg treatment on IHC biomarkers in rectal mucosa by *TRPM7* genotype

Compared with the placebo arm, reducing the Ca:Mg intake ratio to 2.3 through Mg supplementation did not significantly affect the levels of necroptosis biomarkers (i.e., TRPM7 and pMLKL), inflammation biomarkers (i.e., COX-2), apoptosis biomarkers (i.e., BAX and TUNEL), and cell proliferation biomarkers (i.e., Ki67), as well as the ratios of Ki67:BAX and the ratio of Ki67:TUNEL in rectal mucosa (Table 1; Supplemental Figure 1). In the stratified analysis by *TRPM7* genotype, decreasing the intake Ca:Mg ratio with Mg supplementation did not significantly change the above-mentioned biomarkers in those with either GA or GG genotypes.

**TABLE 1:** Changes in rectal mucosa immunohistochemical biomarkers by Mg treatment compared with placebo.

Changes (posttreatment − baseline)		By treatment group				*P*
		*n*	Magnesium	*n*	Placebo	
TRPM7	Mean ± SD	112	0.06 ± 1.31	112	−0.03 ± 1.20	0.92
	Median (IQR)		−0.07 (−0.73 to 0.74)		0.03 (−0.60 to 0.58)	
pMLKL	Mean ± SD	112	0.05 ± 0.94	111	0.04 ± 0.90	0.83
	Median (IQR)		0.10 (−0.45 to 0.53)		−0.02 (−0.43 to 0.64)	
TUNEL	Mean ± SD	116	0.15 ± 1.14	112	0.16 ± 1.35	0.88
	Median (IQR)		0.17 (−0.71 to 0.66)		0.10 (−0.71 to 1.05)	
BAX	Mean ± SD	116	−0.13 ± 1.29	112	−0.19 ± 1.24	0.46
	Median (IQR)		−0.13 (−0.79 to 0.82)		−0.26 (−0.73 to 0.42)	
ki67	Mean ± SD	115	−0.02 ± 0.64	111	0.00 ± 0.77	0.80
	Median (IQR)		−0.08 (−0.45 to 0.45)		−0.01 (−0.48 to 0.32)	
COX2	Mean ± SD	111	0.08 ± 0.74	109	−0.04 ± 0.82	0.20
	Median (IQR)		0.11 (−0.43 to 0.62)		0.01 (−0.63 to 0.50)	
ki67: TUNEL	Mean ± SD	110	−0.21 ± 1.33	110	−0.23 ± 1.50	0.80
	Median (IQR)		−0.10 (−1.07 to 0.78)		−0.10 (−1.18 to 0.72)	
ki67: BAX	Mean ± SD	115	0.15 ± 1.43	111	0.20 ± 1.33	0.66
	Median (IQR)		0.14 (−0.62 to 0.86)		0.19 (−0.54 to 0.83)	

Values are in log scales. *P* values were calculated using Kruskal–Wallis tests comparing the mean changes between randomization groups.

Abbreviations: BAX, BCL2-associated X; COX, cyclooxygenase; IHC, immunohistochemical; pMLKL, phosphorylated mixed lineage kinase like; TRPM7, transient receptor potential melastatin 7; TUNEL, terminal deoxynucleotidyl transferase dUTP nick end labeling.

### Associations between the *TRPM7* genotype and the gut microbiota

**FIGURE 2:**
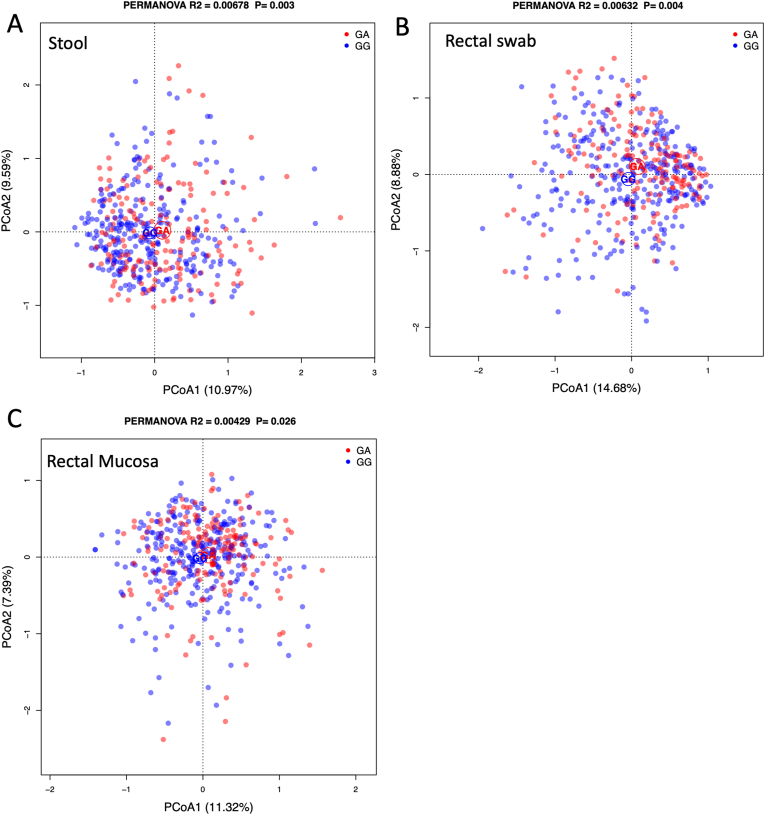
Multidimensional scaling of genus composition of stool, rectal swab, and rectal mucosa tissue samples of study participants. Stool (A), swab (B), and tissue (C) samples were colored based on genotype. The ellipses indicate the 95% confidence limits of each genotype. PCoA, principal coordinate analysis; PERMANOVA, permutational multivariate ANOVA.

We analyzed the associations between the *TRPM7* genotype and the relative abundances of gut taxa as measured in stools, rectal swabs, and rectal mucosal tissues. We first analyzed the microbial association with the genotype at the microbial community level using the PERMANOVA test and found that the *TRPM7* genotype was significantly associated with taxonomic variation of the individuals’ gut microbiota in all 3 sample types with a modest effect size (stool—*R*^2^ = 0.0068; *P* = 0.003; swab—*R*^2^ = 0.0063; *P* = 0.004; tissue—*R*^2^ = 0.0043; *P* = 0.026) (Figure 2). We used PCoA to visualize the differences between genotypes and found that the 95% confidence limits of the 2 genotypes were not separated at the first 2 PCoA axes except for swab samples. We also observed similar separations between the GG and GA genotypes in pretreatment and posttreatment groups with the PCoA in stool and swab samples (Supplemental Figure 2). However, when we analyzed the microbial association with the genotype in the pretreatment and posttreatment groups separately with PERMANOVA tests, the *R*^2^ remained similar but the *P* values were no longer significant (Supplemental Figure 2). It is likely that the current sample size lacks statistical power, and future studies with a large sample size are needed to verify the findings.

**FIGURE 3:**
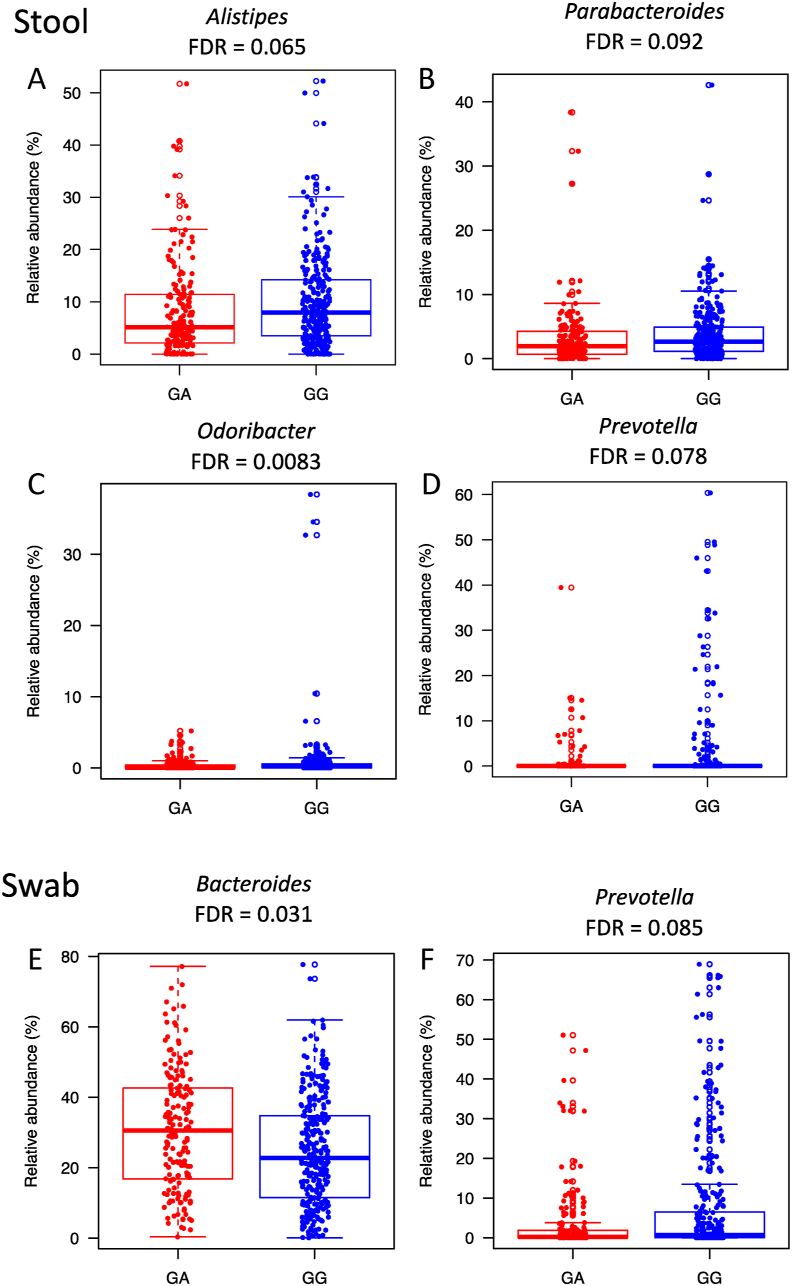
Boxplots of genus compositions that are significantly different between genotypes in stool and rectal swab samples. There were no significantly different genera in rectal mucosa tissue samples (Wilcoxon test, FDR < 0.1). FDR, false discovery rate.

In addition to the PERMANOVA tests examining the *TRPM7* genotype and gut microbiota composition, we analyzed the associations for each taxon and pathway using the univariate nonparametric Wilcoxon test. A total of 14 taxa (phylum to species level) in stools and swabs had significantly different relative abundance between the *TRPM7* genotypes in stools and swabs, but not in tissues after correcting for multiple hypotheses testing [false discovery rate (FDR) < 0.1] (Supplemental Figure 3). The genera significantly associated with *TRPM7* included *Bacteroides, Prevotella, Alistipes, Parabacteroides*, and *Odoribacter*. *Alistipes, Parabacteroides*, and *Odoribacter* in stool samples were higher in individuals with the GG genotype. *Prevotella* had a higher relative abundance in individuals with the GG genotype in both stool and swab samples (Figure 3). *Bacteroides* had a higher relative abundance in swab samples for those with the GA genotype. *Dialister invisus* was more abundant in individuals with the GA genotype in stools and swabs, whereas *Lachnospiraceae* bacterium 3_1_57FAA_CT1 had a higher abundance in individuals with the GA genotype in stools. *Odoribacter splanchnicus* and *Alistipes finegoldii*, and the order *Bacteroidales* were more abundant in individuals with the GG genotype in stool samples. There were no significantly different taxa in tissue samples. We used linear regression models to test the interactions between *TRPM7* genotypes and BMI, and the microbial associations with genotype adjusted for BMI. We found that the interactions were not significant for these taxa in both stool and rectal swab samples. After adjusting for BMI, 8 taxa in stool samples were still significantly associated with genotype, whereas 6 taxa including *Odoribacter*, *Parabacteroides*, *Alistipes*, *Lachnospiraceae* bacterium 3_1_57FAA_CT1, and *Dialister invisus* were not significantly associated. The 5 taxa in rectal swab samples remained significant after adjusting for BMI (Supplemental Table 3).

Similarly, we used PERMANOVA and PCoA to identify potential associations between pathway profiles and the genotype. We analyzed both the pathway abundance stratified by taxa and the unstratified abundance (the total abundance of each pathway across all taxa) (Supplemental Figures 4 and 5). The associations of individual pathways and genotypes were tested with the Wilcoxon test. The significant pathways were only found in tissue samples after correcting for multiple hypotheses testing, including unstratified PWY-6386: UDP−N−acetylmuramoyl−pentapeptide biosynthesis II, stratified PEPTIDOGLYCANSYN-PWY: peptidoglycan biosynthesis I (meso−diaminopimelate containing) and PWY-6387: UDP−N−acetylmuramoyl−pentapeptide biosynthesis I (meso−diaminopimelate containing) in *Bacteroides stercoris* (Supplemental Figure 6).

### *TRPM7*-related desulfating microbiota and pathways and risk of metachronous colorectal adenoma/serrated polyp

The genera *Bacteroides, Prevotella, Alistipes, Parabacteroides,* and *Odoribacter* were significantly associated with the *TRPM7* genotype in ≥1 sample type. We next asked whether their relative abundances in mucosa, rectal swabs, and stools at the end of the trial were associated with metachronous colorectal adenoma/serrated polyp risks. *Bacteroides* and *Prevotella* were significantly associated with risk of metachronous colorectal polyps (Table 2; Supplemental Figure 7), whereas the other 3 genera, *Alistipes, Parabacteroides,* and *Odoribacter*, were not (Supplemental Table 4). Overall, the abundance of *Prevotella*, but not *Bacteroides*, in rectal mucosa was associated with a nearly 3-fold increased risk of metachronous colorectal polyps (i.e., colorectal adenoma/serrated polyps) in a dose-response manner (*P*-trend = 0.01) after adjusting for potential confounding factors (Table 2). The association remained statistically significant after FDR correction. In the stratified analysis by polyp subtype, we observed that the highest abundance of *Prevotella* in rectal mucosa was associated with a 3.25-fold (95% CI: 1.32, 8.40) increased risk of metachronous adenomas (*P*-trend = 0.01). The association also remained significant after adjusting for multiple comparisons. These associations did not significantly differ by the *TRPM7* genotype (*P*-interaction = 0.50). The highest tertile abundance of *Bacteroides* in swabs was related to an increased risk of metachronous serrated polyps with an odds ratio of 11.04 (95% CI: 1.18, 103.63; *P*-trend = 0.02; *P*-interaction with the *TRPM7* genotype = 0.71), whereas the abundance of *Prevotella* and *Bacteroides* in stool samples was not significantly associated with risk of adenomas and/or serrated polyps. *Alistipes, Parabacteroides*, and *Odoribacter* in mucosa, rectal swabs, and stool samples were not significantly associated with risk of metachronous polyps, regardless of polyp type (Supplemental Table 4). As shown in Supplemental Table 5, the pathway abundance at the end of the trial were not significantly associated with risk of metachronous polyps.

**TABLE 2:** Odds ratios (ORs) and 95% CIs for risk of metachronous colorectal polyps by posttrial levels of bacteria in mucosal tissue and rectal swab.

Bacteria	Cases/controls	Biomarker level (Tertile)			*P*-trend	FDR
		T1	T2	T3		
		Reference (low)	OR (95% CI)	OR (95% CI) (high)		
Rectal mucosa						
Metachronous polyp (adenoma/serrated polyp)						
*g_Bacteroides*	68/53	1.00	0.45 (0.16, 1.30)	1.46 (0.53, 3.97)	0.40	0.48
*g_Prevotella*	68/53	1.00	1.89 (0.10, 35.36)	2.99 (1.10, 8.10)	0.03∗	0.09
Metachronous adenoma						
*g_Bacteroides*	41/53	1.00	0.30 (0.08, 1.14)	1.52 (0.49, 4.77)	0.36	0.48
*g_Prevotella*	41/53	1.00	3.47 (0.16, 76.66)	4.80 (1.43, 16.06)	0.01∗	0.06
Metachronous serrated polyp						
*g_Bacteroides*	14/53	1.00	1.19 (0.21, 6.92)	2.85 (0.50, 16.20)	0.20	0.40
*g_Prevotella*	14/53	1.00	—	1.07 (0.20, 5.65)	0.98	0.98
Rectal swab						
Metachronous polyp (adenoma/serrated polyp)						
*g_Bacteroides*	69/53	1.00	1.21 (0.44, 3.33)	2.18 (0.73, 6.55)	0.16	0.48
*g_Prevotella*	69/53	1.00	0.80 (0.29, 2.17)	1.14 (0.36, 3.58)	0.83	0.89
Metachronous adenoma						
*g_Bacteroides*	42/53	1.00	0.90 (0.27, 2.95)	1.10 (0.31, 3.96)	0.88	0.89
*g_Prevotella*	42/53	1.00	0.80 (0.24, 2.69)	1.80 (0.44, 7.40)	0.41	0.82
Metachronous serrated polyp						
*g_Bacteroides*	14/53	1.00	5.97 (0.41, 87.35)	38.94 (1.91, 792.16)	0.009∗∗	0.05
*g_Prevotella*	14/53	1.00	—	0.93 (0.17, 5.17)	0.89	0.89
Stool						
Metachronous polyp (adenoma/serrated polyp)						
*g_Bacteroides*	68/50	1.00	1.26 (0.47, 3.38)	1.03 (0.36, 2.93)	0.94	0.94
*g_Prevotella*	68/50	1.00	1.20 (0.40, 3.61)	1.62 (0.50, 5.26)	0.43	0.80
Metachronous adenoma						
*g_Bacteroides*	41/50	1.00	1.41 (0.44, 4.50)	1.90 (0.55, 6.53)	0.31	0.80
*g_Prevotella*	41/50	1.00	1.10 (0.30, 4.06)	1.08 (0.26, 4.45)	0.90	0.94
Metachronous serrated polyp						
*g_Bacteroides*	14/50	1.00	0.96 (0.21, 4.49)	0.57 (0.10, 3.17)	0.53	0.80
*g_Prevotella*	14/50	1.00	—	1.89 (0.34, 10.36)	0.41	0.80

Unconditional logistic regression models adjusted for age (continuous), sex, BMI (continuous) and baseline level. Tests for trend across tertiles were performed in logistic regression models by assigning1, 2, and 3 to the levels of bacteria (*g*_ indicates genus level). Due to small sample sizes for metachronous serrated polyp, there was no cases in the middle tertile for *g*_*Prevotella*.

∗*P* < 0.05; ∗∗*P* < 0.01.

### Effects of Mg treatment on the abundance of *TRPM7-*related tumorigenic microbes

*Prevotella* and *Bacteroides* are among the most prominent colorectal cancer–associated bacteria, reported in higher abundance in cancer than those in normal tissues [41,42]. *Prevotella* and *Bacteroides* are mucin-degrading bacteria [18], and they possess desulfating capacities to desulfate glycans from host and diets [[43], [44], [45]]. Since desulfating enzymes are dependent on Ca:Mg ratios, we hypothesized that compared with the placebo arm, reducing intake Ca:Mg ratios to 2.3 with Mg supplementation may reduce the abundance of *Prevotella* and *Bacteroides*. We conducted the analyses in rectal mucosa, rectal swabs, and stool samples (Table 3). Optimizing the Ca:Mg intake ratio to 2.3 through Mg supplementation did not significantly change the abundance of *Prevotella* and *Bacteroides* in rectal mucosa but reduced *Prevotella* by 13.4% in rectal swabs (*P* = 0.06) and *Bacteroides* by 1.3% in stool samples (*P* = 0.03), respectively, compared with placebo. In stratified analysis by the *TRPM7* genotype, we found that optimizing the Ca:Mg intake ratio to 2.3 through Mg supplementation did not change *Prevotella* and *Bacteroides* abundance in mucosa regardless of *TRPM7* genotype (*P*-interaction = 0.64). On the contrary, compared with the placebo arm, decreasing the intake Ca:Mg ratios to 2.3 with Mg supplementation reduced the abundance of *Prevotella* by 24.8% in rectal swabs (*P* = 0.007) and *Bacteroides* by 1.7% in stool samples (*P* = 0.03) in those with the GA genotype, but no significant effects were found in those with the GG genotype. The interaction between the *TRPM7* genotype and abundance of *Prevotella* in rectal swabs was statistically significant (*P*-interaction = 0.03).

**TABLE 3:** Changes in *Prevotella* and *Bacteroides* by Mg treatment compared with placebo in rectal mucosa, swab, and stool.

Bacteria	Change from baseline				*P*_1_	*P*_2_
	Treatment	Change (%)	Placebo	Change (%)		
Total (*n* = 229)						
Rectal mucosa						
g_B*acteroides*	−0.005 ± 0.208	−0.08	−0.026 ± 0.232	−0.41	0.47	0.51
g_*Prevotella*	0.042 ± 2.291	2.03	0.203 ± 2.457	9.87	0.61	0.59
Rectal swab						
g_B*acteroides*	−0.000 ± 0.392	−0.01	−0.014 ± 0.406	−0.23	0.79	0.40
g_*Prevotella*	−0.450 ± 2.057	−13.37	0.074 ± 2.195	1.97	0.06	0.06
Stool						
g_B*acteroides*	−0.078 ± 0.483	−1.25	0.046 ± 0.412	0.74	0.04∗	0.03∗
g_*Prevotella*	0.095 ± 1.577	5.55	0.052 ± 1.743	3.05	0.85	0.88
*TRPM7* genotype: GG (*n* = 143)						
Rectal mucosa						
g_B*acteroides*	−0.030 ± 0.205	−0.48	−0.027 ± 0.252	−0.42	0.93	0.79
g_*Prevotella*	0.108 ± 2.268	5.02	0.245 ± 2.686	12.13	0.74	0.84
Rectal swab						
g_B*acteroides*	0.024 ± 0.413	0.39	−0.013 ± 0.465	−0.21	0.61	0.24
g_*Prevotella*	−0.174 ± 1.954	−4.24	−0.095 ± 2.239	−2.36	0.82	0.83
Stool						
g_B*acteroides*	−0.063 ± 0.536	−1.01	0.022 ± 0.410	0.35	0.29	0.24
g_*Prevotella*	−0.059 ± 1.716	−2.90	−0.001 ± 1.814	−0.04	0.84	0.83
*TRPM7* genotype: GA (*n* = 86)						
Rectal mucosa						
g_B*acteroides*	0.039 ± 0.208	0.61	−0.025 ± 0.196	−0.39	0.15	0.07
g_*Prevotella*	−0.076 ± 2.355	−4.05	0.132 ± 2.034	6.20	0.67	0.54
Rectal swab						
g_B*acteroides*	−0.041 ± 0.355	−0.66	−0.017 ± 0.283	−0.27	0.72	0.77
g_*Prevotella*	−0.919 ± 2.165	−24.84	0.364 ± 2.111	10.84	0.007∗∗	0.007∗∗
Stool						
g_B*acteroides*	−0.105 ± 0.377	−1.68	0.084 ± 0.418	1.37	0.03∗	0.04∗
g_*Prevotella*	0.363 ± 1.276	32.40	0.137 ± 1.639	8.85	0.48	0.68

Data are given as mean ± SD. Generalized linear model were used: *P*_1_ was not adjusted; *P*_2_ was adjusted for age, sex, BMI, and baseline level. *P* values reflect tests comparing the mean changes between randomization groups.

∗*P* < 0.05; ∗∗*P* < 0.01.

### Untargeted analysis to identify tumorigenic microbes linked to risk of metachronous colorectal adenoma/serrated polyp independent of genotype

Finally, we conducted an untargeted analysis in addition to the aforementioned *TRPM7* genotype–linked microbes to screen the associations of metachronous adenoma/serrated polyps with all nonrare genera (present in >25% samples) and species identified by shotgun metagenomics. After FDR correction, we found 6 taxa in stool samples significantly associated with metachronous colorectal adenoma/serrated polyps, 12 significant taxa in swab samples, and 3 significant taxa in tissue samples (Table 4; Supplemental Figure 8). There were 4 taxa in stool, 5 in swab, and 3 in tissue, which were significantly associated with adenoma, whereas 9 taxa were significantly associated with serrated polyps in rectal swab samples and 1 in stool samples. In stool samples, the associated taxa were mainly *Barnesiella* spp, *Roseburia* spp, and *Bacteroides* spp, with higher abundance in metachronous polyp cases. In rectal swab samples, *Barnesiella* spp, *Bacteroides* spp, and *Acidaminococcus* spp were more abundant in cases, whereas *Eggerthella, Clostridium*, *Anaerotruncus,* and *Flavonifractor* spp were more abundant in controls. In rectal mucosa, the abundance of *Bacteroides faecis* and *Acidaminococcus* species were higher in cases, and the abundance of *Ruminococcus torques* and *Blautia* sp was lower in cases than that in controls. The taxa significantly associated with metachronous adenoma/serrated polyps were different across the 3 sampling methods. There were 13 taxa unique to rectal swabs, 3 to rectal tissues, and 4 to stool samples (Table 4). Three taxa were significant in both stool and swabs, 1 taxon in both stool and tissue, and 2 in both swabs and tissues.

**TABLE 4:** Taxa significantly different between^1^ metachronous colorectal polyps and controls in stool, rectal swab, and rectal mucosa microbiota in the untargeted analyses.

	Stool only	Swab only	Tissue only	Stool and swab	Stool and tissue	Swab and tissue	All
Polyps	*g_Roseburia* ↑ *s_Roseburia inulinivorans*↑	*g_Eggerthella* ↓ *s_Eggerthella* unclassified ↓ *s_Prevotella copri*^2^ ↑ *g_Clostridium* ↓ *s_Clostridium symbiosum* ↓ *g_Anaerotruncus* ↓ *s_Anaerotruncus colihominis* ↓	none	*s_Bacteroides dorei* ↑ *g_Barnesiella* ↑ *s_Barnesiella intestinihominis* ↑	*s_Bacteroides faecis* ↑	*g_Acidaminococcus* ↑ *s_Acidaminococcus* unclassified ↑	None
Adenoma	*g_Roseburia* ↑ *s_Roseburia intestinalis* ↑	*s_Prevotella copri*^2^ ↑ *g_Anaerotruncus* ↓ *s_Anaerotruncus colihominis* ↓	*s_Bacteroides faecis*↑ *s_Ruminococcus torques* ↓	*g_Barnesiella* ↑ *s_Barnesiella intestinihominis* ↑	None	None	None
Serrated polyps	*s_Parabacteroides* unclassified ↓	*g_Eggerthella* ↓ *s_Eggerthella* unclassified ↓ *g_Bacteroides* ↑ *s_Bacteroides dorei*^2^ ↑ *g_Clostridium* ↓ *g_Flavonifractor* ↓ *s_Flavonifractor plautii* ↓ *s_Lachnospiraceae bacterium 3 1 57FAA CT1* ↓ *s_Roseburia inulinivorans* ↑	None	None	None	None	None

1 Taxa were considered as significant with FDR < 0.1. ↑ indicates significantly increased abundance in cases compared with that in controls, whereas ↓ represents significantly reduced abundance in cases than that in controls. *g*_ indicates genus level and *s*_ species level.

2 Based on the latest nomenclature in National Center for Biotechnology Information (NCBI) taxonomy (January 2025), *Prevotella copri* is now named *Segatella copri*, and *Bacteroides dorei* is now named *Phocaeicola dorei*. The original nomenclatures are still used so the findings can be compared with other literatures where the original nomenclatures have been widely used.

Consistent with our findings for the *TRPM7*-associated microbiota (genera *Prevotella* and *Bacteroides*), in the untargeted analysis, we also found that *Prevotella* spp. (*Prevotella copri*), genus *Bacteroides*, and *Bacteroides* spp. (*B faecis* and *Bacteroides dorei*) were positively associated with metachronous adenoma/serrated polyp.

### Effects of Mg treatment on the abundance of tumorigenic microbes identified from the untargeted approach

Next, we evaluated whether compared with the placebo arm, reducing intake Ca:Mg ratios to 2.3 through a personalized Mg treatment improved the abundance of microorganisms linked to risk of metachronous adenomas/serrated polyps using the untargeted approach. Again, we examined the effects in rectal mucosa, rectal swabs, and stool samples (Supplemental Table 6). Mg treatment reduced the abundance of *P copri* and *Acidaminococcus* spp in rectal swabs (*P* < 0.01), but it was not significant after FDR correction. These 2 microbes were associated with increased risk of metachronous polyps in those with the GA genotype of the *TRPM7* gene. Mg treatment also reduced the abundance of *B faecis* in stool samples of patients with the GA genotype (*P* = 0.03), but it was not significant after FDR correction. In an exploratory untargeted analysis screening the changes of all taxa after treatment, no taxa were significant after adjusting for multiple hypotheses testing with an FDR cutoff of 0.1.

## Discussion

In the PPCCT participants with a history colorectal adenoma/serrated of polyp, who consumed diets with the Ca:Mg intake ratio of ≥2.6, optimizing the Ca:Mg intake ratio from over 2.6 to 2.3 through Mg supplementation did not change any of the following biomarkers: necroptosis (pMLKL and TRPM7), inflammation (COX-2), apoptosis (TUNEL and BAX), and cell proliferation (Ki67). The *TRPM7* polymorphism was significantly associated with overall microbiota assemblage in stool, rectal swab, and rectal mucosal samples. Specifically, the *TPRM7* polymorphism was linked to altered relative abundances of *Prevotella* and *Bacteroides* in rectal swabs. Increased abundance of *Prevotella* in rectal mucosa was associated with nearly 3- to 4-fold increased risk of metachronous colorectal polyps, particularly metachronous adenomas. On the contrary, higher abundance of *Bacteroides* in swabs was prospectively linked to increased risk of metachronous serrated polyps. Optimizing the Ca:Mg intake ratio to 2.3 through Mg supplementation reduced the abundance of *Prevotella* in rectal swabs and *Bacteroides* in stool samples compared with the placebo. Furthermore, these effects were only observed in those with the GA genotype.

We used 3 different sample types to estimate the abundances of gut microbiota based on their distances from colorectal mucosa, including stool (the farthest), rectal swab (attached to), and rectal mucosa itself collected for all PPCCT participants [37,38]. Most previous studies have used stool samples and few used tissue samples [46,47]. The colon has the steepest oxygen gradient in the body, with anoxia sharply increasing from the mucosa to the middle of lumen and luminal microbes are more likely to be anaerobic than mucosal communities [37,38]. There is substantial variability in the assessment of the gut microbial community with different sample types [37,38]. Using stool, rectal swab, and rectal mucosa samples, we found multiple microbes from the 3 types of biospecimens were linked to metachronous adenoma/serrated polyps, with the highest number from rectal swabs that have not been widely used in gut microbial research. The rectal swabs are directly attached to gut epithelial cells, which are more responsive to the development of colorectal polyp. In addition, some of the microbes associated with polyp risk were unique in different sampling methods, suggesting that the choice of sampling method should be considered in future research studying the associations between microbiota and polyp risk.

We found that *Prevotella* in rectal mucosa and *Bacteroides* in rectal swabs were associated with metachronous adenomas and serrated polys, respectively. It is consistent with previous findings of cross-sectional studies that *Prevotella* and *Bacteroides* are among the most prominent colorectal cancer-associated bacteria [41,42]. In healthy individuals, a mucus gel layer envelops the mucosa, acting as a barrier that keeps the epithelial surface separated from luminal bacteria across the colon [48]. Sulfation of mucin provides enhanced protection from mucin-degrading bacteria without desulfating capacity [18,23] and reduces inflammation [24]. It was reported that some *Bacteroides* species are equipped with gene clusters termed polysaccharide utilization loci that encode all requisite functions to sensor and metabolize dietary and host mucin glycans [21]. Removing sulfate from sulfated mucin glycans is a rate-limiting process. A mucin-desulfating glycosidase was identified in *Prevotella* (e.g., strain *RS2*), indicating that some *Prevotella* species can desulfate sulfated mucin glycans by directly removing a sulfated sugar from mucin oligosaccharide chains [43,44]. Although *Bacteriodes* can also desulfate mucin glycans, the mucin-degrading power of *Prevotella RS2* [18,23,43,44] was stronger than the protumorigenic *Bacteroides fragilis* [49]. Consistent with this finding, *Prevotella* spp. have been linked to localized and systemic inflammatory diseases and caused a stronger cytokine response than *B fragilis* [46,50]. Colonization of mouse gut microbiota with *Prevotella* spp. increased the host susceptibility to inflammation in mucosa [51]. We hypothesize that *Prevotella* might initiate the desulfation of sulfated mucin glycans in mucosa and the desulfated mucin glycans could be degraded by *Bacteroides* directly attached to mucosa that was sampled by rectal swabs.

Optimizing the Ca:Mg intake ratio to 2.3 from over 2.6 through Mg supplementation in this study significantly reduced the abundance of *Bacteroides* in stool samples and the abundance of *Prevotella* in rectal swabs, primarily among those with the GA genotype. This finding, together with the associations between *Prevotella, Bacteroides*, and risk of metachronous adenoma and serrated polyps, implied the role of gut microbiota in the previously reported association of Ca:Mg intake ratios, *TRPM7* genotype, and risk of adenoma and serrated polyps [7,[29], [30], [31], [32], [33],^52^]. Gut microbiota could mediate the effect of Ca:Mg ratio through altering their bioavailability [53], which may contribute to the association between low Mg level and elevated risk of colorectal cancer [7,[54], [55], [56], [57]]. The desulfating enzymes in *Prevotella* and *Bacteroides* are highly conserved and depend on Ca concentration, but Mg can compete for Ca-binding sites when Mg levels are high [26,27]. Both high Ca:Mg intake ratios and the GA genotype are linked to Mg deficiency [7,[29], [30], [31], [32], [33],^52^], which may enhance the growth of desulfating bacteria in the mucin layer. *Prevotella* in rectal swabs and *Bacteroides* in stool samples were reduced by Mg treatment compared with those by the placebo arm. However, the intervention, which lasted for only 3 mo, may not be long enough to observe changes in other samples, and a longer treatment period is needed for future studies.

Among the taxa significantly associated with metachronous polyps from untargeted analysis, mucosal *Blautia* and *R torques* were less abundant in metachronous adenoma cases than controls. Both *Blautia* sp. and *R torques* are butyrate-producing bacteria [58] and decreased in patients with inflammatory bowel disease [59]. The abundances of *Acidaminococcus* in rectal tissues and rectal swabs and *B faecis* in rectal tissues and stool samples were more abundant in cases with metachronous polyps than those in controls. A recent study found that abundance of *Acidaminococcus* increased significantly in colorectal mucosa but not in stool samples in patients with inflammatory bowel diseases than that in controls [60]. Previous studies also observed that microbes belonging to genus *Acidaminococcus* can use glutamate as the only source of carbon and energy [61]. Glutamate plays an important role in restoring gut barrier function [62,63], and *Acidaminococcus* may hinder the restoration of gut barrier functions through metabolizing glutamate. The abundance of *Roseburia* was significantly associated with polyps and adenoma risks in stool samples. Similar to *Prevotella* and *Bacteroides*, *Roseburia* can degrade mucin and the species *R. inulinivorans* possesses extensive mucolytic functions [64].

The strengths of this study include its randomized, placebo-controlled design to evaluate the effect of optimizing the Ca:Mg intake ratio through Mg supplementation on gut microbiota. With a precision-based design, all background intakes of Mg and Ca from both diet and supplements were measured twice before and 4 times during the treatment, and a personalized dosing strategy of Mg supplementation was provided to each individual and the intake Ca:Mg ratios remained stable in both treatment and placebo arms during the trial. We had high compliance with the study medication, and the dropout rate was very low. In addition, we collected 3 types of gut microbial biospecimens including stool, rectal swabs, and rectal biopsies both before and after treatment.

This current study has limitations. The statistical power may not be sufficient for the analysis although, to our knowledge, this is one of the largest studies on *TRPM7* genotype and microbiota. To address this for microbiome data not meeting the assumption of normal distribution, we used both nonparametric tests and linear models and used both targeted and exploratory untargeted approaches for the analysis and verified if the findings were consistent. We performed genotype-related analysis with both pretreatment and posttreatment time points to increase statistical power and verified that the changes of microbial taxa were consistent with those using only pretreatment time points (Supplemental Figure 9). Future studies with a larger sample size are needed to further verify the findings. This study also focused on the analysis at the microbial genus level because of their higher classification accuracy than that of the species level. Future studies identifying individual species with assembly or culture-based approach will provide more insights into the mechanisms of the microbial associations with *TRPM7* genotype.

In conclusion, the abundance of *Prevotella* and *Bacteroides* was associated with *TRPM7* genotype. Abundance of *Prevotella* in mucosa and *Bacteroides* in rectal swabs was associated with an increased risk of metachronous colorectal adenomas and serrated polyps, respectively. Reducing high Ca:Mg intake ratios to 2.3 through Mg supplementation also reduced the abundance of *Prevotella* in rectal swabs and *Bacteroides* in stool samples. In addition to *Prevotella* and *Bacteroides*, multiple microbes in rectal mucosa, rectal swabs, and/or stool samples were prospectively associated with risk of metachronous adenomas and serrated polyps. Optimizing the Ca:Mg intake ratio through Mg supplementation did not change the host biomarkers related to colorectal carcinogenesis in those who consumed diets with the Ca:Mg intake ratio of ≥2.6. In general, these findings indicate that optimizing the Ca:Mg intake ratio through Mg supplementation may alter the microbiota linked to both the *TRPM7* genotype and risk of metachronous colorectal polyps but not the host response biomarkers (necroptosis and inflammation).

## Author contributions

The authors’ responsibilities were as follows – QD, MJS: contributed to the hypothesis development and the study design; XZ, HJM, RMS, MAA-P, MJS, QD: conducted the research to acquire data; SS: processed the shotgun metagenome sequences for taxonomic and functional profiles and performed the multivariate analyses and the untargeted analysis between gut microbiota and polyp risk; XZ: performed the analyses of demographic characteristic, IHC biomarkers, targeted analysis between gut microbiota and polyp risk, and the effect of Mg treatment; TS: contributed to the IHC assay; XH, CY: contributed to statistical analyses; QD, XZ, SS: drafted the manuscript; and all authors: contributed to writing, review, and/or revision of the manuscript and approved the final manuscript.

## Funding

This study was supported by R01CA149633 and R01CA202936 from the National Cancer Institute; R01DK110166 from National Institute of Diabetes and Digestive and Kidney Diseases, Department of Health and Human Services; and the Ingram Cancer Center Endowment Fund. Data collection, sample storage, and processing for this study were partially conducted by the Survey and Biospecimen Shared Resource, which is supported in part by P30CA068485. Clinical visits to the Vanderbilt at the Clinical Research Center were supported in part by the Vanderbilt CTSA grant UL1 RR024975 from NCRR/NIH. The parent study data were stored in Research Electronic Data Capture (REDCap), and data analyses (VR12960) were supported in part by the Vanderbilt Institute for Clinical and Translational Research (UL1TR000445). The UNC Microbiome Core is funded in part by the Center for Gastrointestinal Biology and Disease (CGIBD P30 DK034987) and the UNC Nutrition Obesity Research Center (NORC P30 DK056350).

## Conflict of interest

The author reports no conflicts of interest.
