# Supplementary material for: The Association of Gut Microbiota With TRPM7 Genotype, Colorectal Polyps, and Magnesium
Source: J Nutr. 2025 Jul 31;155(11):3713–25. doi: 10.1016/j.tjnut.2025.07.015 (PMC12799418; doi:10.1016/j.tjnut.2025.07.015)
Supplement: multimedia component 1 [file mmc1.pdf]

Supplementary Materials

The association of gut microbiota with TRPM7 genotype, colorectal polyps and magnesium

Shan Sun[1*], Xiangzhu Zhu[2*], Xiang Huang[2], Chang Yu[3], Timothy Su[2], Harvey J. Murff[4], Reid M. Ness[5], M. Andrea Azcarate-Peril[6], Martha J. Shrubsole[2], Qi Dai[2]

[1]Department of Bioinformatics and Genomics, University of North Carolina at Charlotte, Charlotte, NC, USA

[2]Division of Epidemiology, Department of Medicine, Vanderbilt University Medical Center, Nashville, TN, USA.

[3]Division of Biostatistics, Department of Population Health at NYU Grossman School of Medicine, New York, NY, USA.

[4]Division of Geriatric Medicine, Department of Medicine, Vanderbilt University Medical Center, Nashville, TN, USA.

[5]Division of Gastroenterology, Hepatology, and Nutrition, Department of Medicine, Vanderbilt University Medical Center, Nashville, TN, USA

[6]Department of Medicine, and Microbiome Core Facility, School of Medicine, University of North Carolina, Chapel Hill NC, USA

[*] Contributed equally

Corresponding author: Qi Dai, Vanderbilt University Medical Center; 2525 West End Ave, Nashville, TN 37203; Phone: 615-936-0707; E-mail: qi.dai@vanderbilt.edu

Supplementary Methods

Whole Genome Shotgun (WGS) sequencing of stool, swab and rectal biopsies

All the samples were frozen at −80 °C until use. Samples were transferred to a 2 ml tube containing 200 mg of ≤106 µm glass beads (Sigma, St. Louis, MO, USA) and 0.3 ml of Qiagen ATL buffer (Qiagen, Valencia, CA, USA), supplemented with lysozyme (20 mg/ml) (Thermo Fisher Scientific, Grand Island, NY, USA). The suspension was incubated at 37°C for 1 h with occasional agitation and then supplemented with 600IU of proteinase K and incubated at 60°C for 1 h. Finally, 0.3 ml of Qiagen AL (Qiagen, Valencia, CA, USA) buffer was added and incubated at 70°C for 10 minutes, followed with 3 min bead beating in a Qiagen TissueLyser II (Qiagen, Valencia, CA, USA) at 30Hz. After a brief centrifugation, supernatants were transferred to a new tube containing 0.3 ml of ethanol. DNA was purified using a standard on-column purification method with Qiagen buffers AW1 and AW2 (Qiagen, Valencia, CA, USA) as washing agents and eluted in 10mM Tris (pH 8.0). 10 ng of genomic DNA was processed using the Illumina Nextera XT DNA Sample Preparation Kit (Illumina, San Diego, CA, USA). Fragmented and tagged DNA was amplified using a limited-cycle PCR program. In this step, index 1 (i7) and index 2 (i5) were added between the downstream bPCR adaptor and the core sequencing library adaptor, as well primer sequences required for cluster formation. The DNA library was purified using Agencourt® AMPure® XP Reagent (Beckman Coulter, Brea, CA). The DNA library pool was loaded on the Illumina platform reagent cartridge and on the Illumina HiSeq instrument (Illumina, San Diego, CA, USA). A blank composed of only DNA isolation reagents was included in the DNA extraction process and again in the library preparation. ZymoBIOMICS Microbial Community DNA Standard (Zymo Research Corporation, Irvine, CA, USA, Cat#D6305) and a library blank composed of library preparation reagents alone were used as controls.

Supplementary Tables

Supplementary Table 1. Primary antibodies and validation data

Antibody	Venter, Catalog no	Host/Isotype	Clone	Ab registry	Applications	Validation of antibody specificity
Anti-TRPM7 [1]	Novus Biologicals, #NBP1-84493	Rabbit	Polyclonal	AB_11001887	IHC	Genetic validation strategies using HEK293 cells demonstrate strong TRPM7 staining, while TRPM7-knockout DT40 cells (DT40 KO) show significantly reduced TRPM7 expression compared to wild-type DT40 cells. The tissue distribution pattern of positive cells is consistent with the Human Protein Atlas dataset.
Anti-pMLKL	Abcam, #ab196436	Rabbit/IgG	EPR9515(2)	AB_2687465	WB, IP	Antibody identity is confirmed at a molecular level for unrivaled batch-batch consistency. It is only detectable in infection/cellular damaged (PMID:29229989) or aging tissue (PMID: 28807105) but not in normal tissues. It specifically detects MLKL Phospho-S345 (UniProt ID: Q9D2Y4; Molecular weight: 55kDa). The tissue distribution pattern of positive cells is consistent with the Human Protein Atlas dataset.
Anti-COX-2	Invitrogen, #18-7379	Mouse/IgG1 kappa	COX 229	AB_2533224	WB, IP, IHC, ELISA	Altered expression of COX-2 upon cell treatment demonstrates antibody specificity. Western blot using this antibody shows increased expression of COX2 in HUVEC upon treatment with 1 ng/mL of IL-1ß for 24 hours. The tissue distribution pattern of positive cells in normal tissues is consistent with the GeneCard database.
Anti-BAX	Cell Signaling, #2774	Rabbit	Polyclonal	AB_490806	WB,IP, IHC-P (rev. 02/08/10)	Cross-reactivity was not detected with other family members at physiological conditions. It detects endogenous levels of total Bax protein. The tissue distribution pattern of positive cells is consistent with the Human Protein Atlas dataset.
Anti-Ki67 [2]	Vector Labs, #VP-K451	Rabbit	Polyclonal	AB_2314701	IHC	Specific nuclear staining. In normal colonic mucosa, the Ki67-positive cells are distributed at the proliferation zone of crypts. The tissue

						distribution pattern of positive cells is consistent with the Human Protein Atlas dataset.

[1] Discontinued. Substituted antibody: Atlas Antibodies, Cat#HPA052173, RRID:AB_2681749
[2] Discontinued. Substituted antibody: Agilent, Cat#M7240, RRID:AB_2142367

Supplementary Table 2. Selected descriptive characteristics of TRPM7 genotypes (Thr1482Ile allele or G->A).

Characteristic	GG	GA	p-value
	152	88	
Age (years)	60.8±8.3	60.8±7.3	0.95
Men, %	85 (55.9)	42 (47.7)	0.22
Education, %			0.59
Under college	14 (9.2)	10 (11.4)	
College or above	138 (90.8)	78 (88.6)	
Income, %			0.61
Below $40,000	22 (14.6)	9 (10.2)	
Equal to $40,000	4 (2.6)	3 (3.4)	
Above $40,000	125 (82.8)	76 (86.4)	
Use of antibiotics in the past 12 months			
Yes	37 (42.0)	24 (33.3)	0.26
Use of NSAIDS			
Yes	54 (35.5)	36 (40.9)	0.41
BMI (kg/m^2)	29.6±5.6	31.4±7.4	0.03
Height (m)	1.7± 0.1	1.7±0.1	0.65
Total energy intake (kcal/day)	2129.6±566.7	2038.6±589.0	0.24

Continuous variables were presented as mean ± SD and *P* values were calculated using Wilcoxon test; categorical variables were presented as frequency (percentage, %) and *P* values were calculated using Pearson chi-square test.
SD: standard deviation

Supplementary Table 3. The association between microbial abundance and TRPM7 genotypes adjusted for BMI in linear regression.

**Taxa in stool samples**	**Genotype P-value**	**Genotype FDR**
p__Bacteroidetes	0.0021	0.0057
c__Bacteroidia	0.0021	0.0057
o__Bacteroidales	0.0021	0.0057
f__Porphyromonadaceae	0.036	0.053
g__Odoribacter	0.10	0.11
s__Odoribacter_splanchnicus	0.0062	0.015
g__Parabacteroides	0.19	0.20
f__Prevotellaceae	0.014	0.030
g__Prevotella	0.034	0.053
f__Rikenellaceae	0.096	0.11
g__Alistipes	0.096	0.11
s__Alistipes_finegoldii	0.020	0.038
s__Lachnospiraceae_bacterium_3_1_57FAA_CT1	0.094	0.11
s__Dialister_invisus	0.76	0.76
**Taxa in rectal swab samples**		
f__Bacteroidaceae	0.00018	0.0017
g__Bacteroides	0.00018	0.0017
f__Prevotellaceae	0.00074	0.0047
g__Prevotella	0.0013	0.0057
s__Dialister_invisus	0.024	0.042

Supplementary Table 4. Odds ratios (ORs) and 95% CIs for risk of metachronous colorectal polyps by post-trial levels of bacteria in mucosal tissue, rectal swab and stool.

Bacteria	Biomarker Level (Tertile)				P_{trend}	*FDR*
	Cases/Controls	REF (Low)	OR (95% CI)	OR (95% CI) (High)		
**Rectal Mucosa**						
	**Metachronous Polyp (adenoma/serrated polyp)**					
g_Alistipes	68/53	1.00	1.67(0.65-4.31)	1.12(0.42-2.98)	0.84	0.84
g_Odoribacter	68/53	1.00	-	-		
g_Parabacteroide	68/53	1.00	1.38(0.37-5.14)	1.10(0.48-2.52)	0.73	0.82
	**Metachronous adenoma**					
g_Alistipes	41/53	1.00	1.04(0.35-3.11)	0.83(0.27-2.54)	0.75	0.82
g_Odoribacter	41/53	1.00	-	-		
g_Parabacteroide	41/53	1.00	1.82(0.42-7.89)	0.64(0.23-1.79)	0.49	0.74
	**Metachronous serrated polyp**					
g_Alistipes	14/53	1.00	3.57(0.59-21.48)	2.43(0.39-15.12)	0.43	0.74
g_Odoribacter	14/53	1.00	-	-		
g_Parabacteroide	14/53	1.00	-	1.33(0.39-4.58)	0.71	0.82
**Rectal swab**						
	**Metachronous Polyp (adenoma/serrated polyp)**					
g_Alistipes	69/53	1.00	1.59(0.62-4.09)	1.47(0.55-3.89)	0.48	0.93
g_Odoribacter	69/53	1.00	0.41(0.13-1.28)	1.22(0.51-2.91)	0.74	0.96
g_Parabacteroide	69/53	1.00	1.42(0.57-3.52)	0.66(0.24-1.84)	0.48	0.93
	**Metachronous adenoma**					
g_Alistipes	42/53	1.00	1.17(0.40-3.45)	1.14(0.37-3.52)	0.82	0.96
g_Dialister	42/53	1.00	0.44(0.11-1.73)	1.41(0.51-3.89)	0.58	0.93
g_Parabacteroide	42/53	1.00	2.47(0.84-7.30)	0.95(0.27-3.36)	0.96	0.96
	**Metachronous serrated polyp**					
g_Alistipes	14/53	1.00	8.47(0.95-75.80)	4.47(0.44-45.34)	0.29	0.93
g_Odoribacter	14/53	1.00		0.66(0.17-2.62)	0.39	0.93
g_Parabacteroide	14/53	1.00	0.57(0.13-2.49)	0.50(0.10-2.42)	0.37	0.93
**Stool**						
	**Metachronous Polyp (adenoma/serrated polyp)**					
g_Alistipes	68/50	1.00	0.80(0.30-2.10)	0.86(0.32-2.32)	0.77	0.99
g_Odoribacter	68/50	1.00	1.23(0.47-3.21)	0.96(0.37-2.49)	0.95	0.99
g_Parabacteroide	68/50	1.00	1.89(0.71-5.03)	1.27(0.44-3.64)	0.69	0.99
	**Metachronous adenoma**					
g_Alistipes	41/50	1.00	0.72(0.23-2.26)	0.81(0.25-2.58)	0.72	0.99
g_Odoribacter	41/50	1.00	1.48(0.49-4.47)	1.01(0.33-3.06)	0.96	0.99
g_Parabacteroide	41/50	1.00	0.99(0.32-3.01)	1.01(0.31-3.26)	0.99	0.99
	**Metachronous serrated polyp**					
g_Alistipes	14/50	1.00	1.09(0.23-5.20)	1.68(0.35-8.07)	0.52	0.99
g_Odoribacter	14/50	1.00	1.27(0.29-5.59)	0.70(0.14-3.40)	0.68	0.99
g_Parabacteroide	14/50	1.00	10.22(1.07-98.07)	5.10(0.48-53.86)	0.28	0.99

Unconditional logistic regression models adjusted for age (continuous), sex, and BMI (continuous).

Supplementary Table 5. Odds ratios (ORs) and 95% CIs for risk of metachronous colorectal polyps by post-trial levels of pathway in rectal swab and stool.

Bacteria	Biomarker Level (Tertile)				P_{trend}
	Cases/Controls	REF (Low)	OR (95% CI)	OR (95% CI) (High)	
**Rectal swab**					
	**Metachronous Polyp (adenoma/serrated polyp)**				
Peptidoglycansyn-PWY	69/53	1.00	0.45(0.13-1.57)	0.66(0.28-1.57)	0.29
PWY-6387	69/53	1.00	0.45(0.14-1.47)	0.64(0.27-1.54)	0.27
PWY-6386	69/53	1.00	1.16(0.46-2.88)	0.62(0.24-1.62)	0.39
	**Metachronous adenoma**				
Peptidoglycansyn-PWY	42/53	1.00	0.14(0.02-0.86)	0.57(0.20-1.61)	0.18
PWY-6387	42/53	1.00	0.09(0.01-0.59)	0.53(0.19-1.51)	0.13
PWY-6386	42/53	1.00	1.24(0.44-3.51)	0.39(0.12-1.33)	0.20
	**Metachronous serrated polyp**				
Peptidoglycansyn-PWY	14/53	1.00	1.52(0.30-7.58)	0.88(0.21-3.66)	0.92
PWY-6387	14/53	1.00	1.18(0.24-5.84)	0.83(0.20-3.43)	0.83
PWY-6386	14/53	1.00	0.61(0.13-2.82)	0.53(0.12-2.48)	0.39
**Stool**					
	**Metachronous Polyp (adenoma/serrated polyp)**				
Peptidoglycansyn-PWY	68/50	1.00	0.39(0.13-1.15)	0.79(0.32-1.93)	0.53
PWY-6387	68/50	1.00	0.39(0.13-1.15)	0.79(0.32-1.93)	0.53
PWY-6386	68/50	1.00	1.24(0.36-4.27)	0.49(0.19-1.26)	0.18
	**Metachronous adenoma**				
Peptidoglycansyn-PWY	41/50	1.00	0.39(0.11-1.39)	0.77(0.27-2.17)	0.56
PWY-6387	41/50	1.00	0.39(0.11-1.39)	0.77(0.27-2.17)	0.56
PWY-6386	41/50	1.00	1.53(0.40-5.93)	0.34(0.10-1.15)	0.14
	**Metachronous serrated polyp**				
Peptidoglycansyn-PWY	14/50	1.00	0.16(0.02-1.63)	1.19(0.32-4.43)	0.88
PWY-6387	14/50	1.00	0.16(0.02-1.63)	1.19(0.32-4.43)	0.88
PWY-6386	14/50	1.00	0.71(0.07-7.19)	0.85(0.22-3.36)	0.80

Unconditional logistic regression models adjusted for age (continuous), sex, and BMI (continuous)
Only a very few rectal mucosa samples have been detected the top five pathways expression.
PEPTIDOGLYCANSYN-PWY: peptidoglycan biosynthesis I (meso-diaminopimelate containing) |g__Bacteroides.s__Bacteroides; PWY-6387: UDP-N-acetylmuramoyl-pentapeptide biosynthesis I (meso-diaminopimelate containing) |g__Bacteroides.s__Bacteroides_stercoris; PWY-6386: UDP-N-acetylmuramoyl-pentapeptide biosynthesis II (lysine-containing).

Supplementary Table 6. Effects of Mg treatment on Abundance of Tumorigenic Microbes Identified from the Unbiased Approach in the PPCCT.

Bacteria	Treatment	Placebo	*P*1	*P*2	*FDR_P2*
Total (n=229)					
**Rectal Mucosa**					
g_Eggerthella	-0.041±0.439	-0.006±0.580	0.60	0.33	0.84
g_Eggerthella|s_Eggerthella_unclassified	-0.041±0.436	-0.006±0.580	0.60	0.33	0.84
g_Bacteroides|s_Bacteroides_dorei	0.251±2.331	-0.029±2.220	0.36	0.54	0.84
g_Bacteroides|s_Bacteroides_faecis	0.002±0.622	0.037±0.992	0.75	0.46	0.84
g_Barnesiella	-0.232±1.783	0.016±2.186	0.35	0.18	0.82
g_Barnesiella|s_Barnesiella_intestinihominis	-0.232±1.783	0.016±2.186	0.35	0.18	0.82
g_Parabacteroides|s_Parabacteroides_unclassified	0.227±2.414	0.592±1.884	0.20	0.83	0.95
g_Prevotella|s_Prevotella_copri	0.078±2.170	0.209±2.620	0.68	0.62	0.90
g_Clostridium	-0.088±1.753	-0.271±1.929	0.46	0.11	0.72
g_Clostridium|s_Clostridium_symbiosum	0.055±0.816	0.023±0.705	0.75	0.65	0.90
g_Flavonifractor	0.011±0.783	0.069±0.763	0.58	0.88	0.95
g_Flavonifractor|s_Flavonifractor_plautii	0.011±0.783	0.069±0.763	0.58	0.88	0.95
g_Blautia	0.123±3.300	0.313±2.812	0.64	0.92	0.95
g_Blautia|s_Ruminococcus_torques	0.128±3.308	0.156±2.852	0.95	0.61	0.89
g_Lachnospiraceae_noname|s_Lachnospiraceae_bacterium_3_1_57FAA_CT1	-0.182±1.454	0.022±1.099	0.23	0.68	0.90
g_Roseburia	0.327±2.434	-0.022±2.174	0.26	0.39	0.84
g_Roseburia|s_Roseburia_intestinalis	0.224±1.500	0.084±1.262	0.45	0.40	0.84
g_Roseburia|s_Roseburia_inulinivorans	0.040±1.193	0.197±1.116	0.31	0.64	0.90
g_Anaerotruncus	0.126±1.149	0.101±0.945	0.85	0.60	0.89
g_Anaerotruncus|s_Anaerotruncus_colihominis	0.025±0.868	-0.002±0.548	0.77	0.40	0.84
g_Acidaminococcus	0.235±1.646	0.278±1.870	0.85	0.40	0.84
g_Acidaminococcus|s_Acidaminococcus_unclassified	0.235±1.646	0.315±1.818	0.73	0.37	0.84
**Rectal Swab**					
g_Eggerthella	0.043±1.973	0.177±2.142	0.62	0.24	0.84
g_Eggerthella|s_Eggerthella_unclassified	0.042±1.971	0.153±2.122	0.68	0.29	0.84
g_Bacteroides|s_Bacteroides_dorei	-0.080±1.230	-0.069±1.214	0.95	0.95	0.96
g_Bacteroides|s_Bacteroides_faecis	0.185±1.334	0.090±1.270	0.58	0.70	0.91
g_Barnesiella	-0.009±0.871	-0.112±1.545	0.53	0.43	0.84
g_Barnesiella|s_Barnesiella_intestinihominis	-0.009±0.871	-0.112±1.545	0.53	0.43	0.84

g_Parabacteroides|s_Parabacteroides_unclassified	0.135±2.298	0.183±1.879	0.86	0.94	0.95
g_Prevotella|s_Prevotella_copri	-0.156±1.380	0.159±1.471	0.09	0.09	0.63
g_Clostridium	0.020±2.052	-0.073±2.234	0.74	0.91	0.95
g_Clostridium|s_Clostridium_symbiosum	-0.088±1.984	0.137±2.051	0.40	0.11	0.72
g_Flavonifractor	0.431±2.306	-0.112±2.305	0.07	0.22	0.83
g_Flavonifractor|s_Flavonifractor_plautii	0.431±2.306	-0.112±2.305	0.07	0.22	0.83
g_Blautia	0.237±2.169	-0.097±1.969	0.22	0.39	0.84
g_Blautia|s_Ruminococcus_torques	0.234±2.356	-0.006±2.424	0.44	0.49	0.84
g_Lachnospiraceae_noname|s_Lachnospiraceae_bacterium_3_1_57FAA_CT1	-0.283±2.138	0.157±2.335	0.13	0.16	0.78
g_Roseburia	0.103±1.642	-0.135±1.748	0.29	0.48	0.84
g_Roseburia|s_Roseburia_intestinalis	0.346±2.611	-0.393±2.477	0.03*	0.03*	0.45
g_Roseburia|s_Roseburia_inulinivorans	0.242±1.887	0.010±2.159	0.38	0.73	0.93
g_Anaerotruncus	0.147±2.349	0.011±2.441	0.66	0.50	0.84
g_Anaerotruncus|s_Anaerotruncus_colihominis	0.217±2.021	0.133±2.217	0.76	0.61	0.89
g_Acidaminococcus	-0.189±1.102	0.041±1.278	0.14	0.05	0.45
g_Acidaminococcus|s_Acidaminococcus_unclassified	-0.159±1.059	0.146±1.294	0.05	0.02*	0.45
**Stool**					
g_Eggerthella	0.198±1.480	-0.054±1.598	0.22	0.26	0.84
g_Eggerthella|s_Eggerthella_unclassified	0.205±1.471	-0.093±1.542	0.13	0.15	0.77
g_Bacteroides|s_Bacteroides_dorei	-0.048±1.040	-0.080±1.335	0.84	0.90	0.95
g_Bacteroides|s_Bacteroides_faecis	-0.182±1.159	0.132±1.105	0.04*	0.04*	0.45
g_Barnesiella	-0.156±1.144	-0.104±1.308	0.75	0.84	0.95
g_Barnesiella|s_Barnesiella_intestinihominis	-0.156±1.144	-0.104±1.308	0.75	0.84	0.95
g_Parabacteroides|s_Parabacteroides_unclassified	0.379±1.933	0.014±1.822	0.14	0.09	0.63
g_Prevotella|s_Prevotella_copri	-0.100±1.335	0.181±1.522	0.14	0.14	0.77
g_Clostridium	-0.067±1.356	0.013±1.606	0.68	0.45	0.84
g_Clostridium|s_Clostridium_symbiosum	-0.080±1.594	0.077±1.765	0.48	0.14	0.77
g_Flavonifractor	0.069±1.688	-0.028±1.659	0.66	0.94	0.95
g_Flavonifractor|s_Flavonifractor_plautii	0.069±1.688	-0.028±1.659	0.66	0.94	0.95
g_Blautia	0.123±1.078	0.076±1.057	0.74	0.77	0.95
g_Blautia|s_Ruminococcus_torques	0.029±1.463	0.104±1.586	0.71	0.55	0.84
g_Lachnospiraceae_noname|s_Lachnospiraceae_bacterium_3_1_57FAA_CT1	-0.107±1.731	0.209±1.984	0.20	0.05	0.45
g_Roseburia	-0.087±0.889	-0.099±1.161	0.93	0.65	0.90

g_Roseburia|s_Roseburia_intestinalis	-0.002±1.910	-0.153±1.948	0.56	0.15	0.77
g_Roseburia|s_Roseburia_inulinivorans	-0.100±1.838	0.123±1.942	0.37	0.13	0.77
g_Anaerotruncus	0.393±2.007	0.032±2.241	0.20	0.37	0.84
g_Anaerotruncus|s_Anaerotruncus_colihominis	0.238±1.539	0.015±1.793	0.31	0.30	0.84
g_Acidaminococcus	-0.013±1.288	-0.189±1.332	0.31	0.63	0.90
g_Acidaminococcus|s_Acidaminococcus_unclassified	0.010±1.415	-0.110±1.204	0.49	0.85	0.95
***TRPM7 genotype: GG* (n=143)**					
	**Rectal Mucosa**				
g_Eggerthella	0.000±0.000	-0.065±0.550	0.32		
g_Eggerthella|s_Eggerthella_unclassified	0.000±0.000	-0.065±0.550	0.32		
g_Bacteroides|s_Bacteroides_dorei	0.183±2.164	0.115±2.270	0.86	0.93	0.95
g_Bacteroides|s_Bacteroides_faecis	0.000±0.000	0.051±0.563	0.44	0.35	0.84
g_Barnesiella	-0.488±1.708	-0.348±1.736	0.63	0.53	0.84
g_Barnesiella|s_Barnesiella_intestinihominis	-0.488±1.708	-0.348±1.736	0.63	0.53	0.84
g_Parabacteroides|s_Parabacteroides_unclassified	0.342±2.510	0.470±1.902	0.73	0.73	0.93
g_Prevotella|s_Prevotella_copri	-0.068±2.143	0.334±2.542	0.31	0.40	0.84
g_Clostridium	-0.145±1.784	-0.590±1.902	0.15	0.06	0.51
g_Clostridium|s_Clostridium_symbiosum	0.004±0.030	-0.074±0.591	0.27	0.44	0.84
g_Flavonifractor	-0.056±0.473	-0.007±0.665	0.61	0.35	0.84
g_Flavonifractor|s_Flavonifractor_plautii	-0.056±0.473	-0.007±0.665	0.61	0.35	0.84
g_Blautia	0.044±3.422	0.259±2.967	0.69	0.92	0.95
g_Blautia|s_Ruminococcus_torques	0.116±3.482	0.075±3.069	0.94	0.72	0.93
g_Lachnospiraceae_noname|s_Lachnospiraceae_bacterium_3_1_57FAA_CT1	-0.056±1.304	-0.081±1.182	0.91	0.55	0.84
g_Roseburia	0.545±2.250	-0.046±2.030	0.10	0.08	0.63
g_Roseburia|s_Roseburia_intestinalis	0.249±1.412	0.124±0.737	0.51	0.25	0.84
g_Roseburia|s_Roseburia_inulinivorans	-0.006±1.003	0.006±0.795	0.94	0.80	0.95
g_Anaerotruncus	0.186±1.197	0.159±1.188	0.89	0.90	0.95
g_Anaerotruncus|s_Anaerotruncus_colihominis	0.027±0.741	-0.003±0.692	0.80	0.86	0.95
g_Acidaminococcus	0.312±1.820	0.235±1.789	0.80	0.53	0.84
g_Acidaminococcus|s_Acidaminococcus_unclassified	0.312±1.820	0.295±1.705	0.95	0.51	0.84
	**Rectal Swab**				
g_Eggerthella	0.053±1.920	-0.102±2.160	0.65	0.51	0.84
g_Eggerthella|s_Eggerthella_unclassified	0.054±1.916	-0.100±2.154	0.65	0.51	0.84

g_Bacteroides|s_Bacteroides_dorei	-0.084±1.228	0.085±1.235	0.41	0.60	0.89
g_Bacteroides|s_Bacteroides_faecis	0.267±1.280	0.100±1.262	0.43	0.36	0.84
g_Barnesiella	-0.031±0.965	-0.252±1.557	0.30	0.20	0.82
g_Barnesiella|s_Barnesiella_intestinihominis	-0.031±0.965	-0.252±1.557	0.30	0.20	0.82
g_Parabacteroides|s_Parabacteroides_unclassified	0.286±2.278	0.050±2.104	0.51	0.52	0.84
g_Prevotella|s_Prevotella_copri	-0.093±1.472	-0.097±1.543	0.99	0.83	0.95
g_Clostridium	0.340±2.289	-0.276±2.356	0.11	0.25	0.84
g_Clostridium|s_Clostridium_symbiosum	0.072±2.158	0.068±2.232	0.99	0.61	0.89
g_Flavonifractor	0.629±2.166	-0.244±2.506	0.03*	0.05	0.45
g_Flavonifractor|s_Flavonifractor_plautii	0.629±2.166	-0.244±2.506	0.03*	0.05	0.45
g_Blautia	0.398±2.070	-0.178±2.313	0.11	0.02*	0.45
g_Blautia|s_Ruminococcus_torques	0.552±2.299	-0.148±2.729	0.10	0.01*	0.39
g_Lachnospiraceae_noname|s_Lachnospiraceae_bacterium_3_1_57FAA_CT1	0.035±2.175	0.093±2.354	0.88	0.89	0.95
g_Roseburia	-0.029±1.551	-0.100±1.942	0.81	0.54	0.84
g_Roseburia|s_Roseburia_intestinalis	0.541±2.409	-0.499±2.554	0.01*	0.01*	0.39
g_Roseburia|s_Roseburia_inulinivorans	0.417±1.883	0.011±2.270	0.24	0.61	0.89
g_Anaerotruncus	-0.045±2.376	-0.099±2.577	0.89	0.50	0.84
g_Anaerotruncus|s_Anaerotruncus_colihominis	0.231±1.935	0.022±2.388	0.56	0.49	0.84
g_Acidaminococcus	-0.027±1.010	-0.026±1.222	1.00	0.88	0.95
g_Acidaminococcus|s_Acidaminococcus_unclassified	-0.035±1.009	0.072±1.066	0.53	0.50	0.84
		**Stool**			
g_Eggerthella	0.095±1.581	-0.216±1.622	0.25	0.50	0.84
g_Eggerthella|s_Eggerthella_unclassified	0.106±1.571	-0.258±1.566	0.17	0.36	0.84
g_Bacteroides|s_Bacteroides_dorei	0.063±1.171	0.000±1.371	0.77	0.69	0.91
g_Bacteroides|s_Bacteroides_faecis	-0.210±1.328	-0.007±1.080	0.32	0.34	0.84
g_Barnesiella	-0.122±1.224	-0.306±1.441	0.41	0.33	0.84
g_Barnesiella|s_Barnesiella_intestinihominis	-0.122±1.224	-0.306±1.441	0.41	0.33	0.84
g_Parabacteroides|s_Parabacteroides_unclassified	0.501±2.046	-0.162±1.740	0.04*	0.02*	0.45
g_Prevotella|s_Prevotella_copri	-0.023±1.361	0.053±1.644	0.76	0.78	0.95
g_Clostridium	-0.052±1.579	-0.069±1.698	0.95	0.53	0.84
g_Clostridium|s_Clostridium_symbiosum	-0.014±1.801	0.083±1.923	0.76	0.16	0.78
g_Flavonifractor	0.248±1.764	-0.108±1.593	0.21	0.54	0.84
g_Flavonifractor|s_Flavonifractor_plautii	0.248±1.764	-0.108±1.593	0.21	0.54	0.84

g_Blautia	0.043±1.074	0.086±0.951	0.80	0.46	0.84
g_Blautia|s_Ruminococcus_torques	0.018±1.354	0.039±1.536	0.93	0.63	0.90
g_Lachnospiraceae_noname|s_Lachnospiraceae_bacterium_3_1_57FAA_CT1	0.100±1.906	0.257±2.205	0.65	0.19	0.82
g_Roseburia	-0.145±0.792	-0.089±1.303	0.76	0.71	0.92
g_Roseburia|s_Roseburia_intestinalis	0.091±1.968	-0.279±1.990	0.27	0.15	0.77
g_Roseburia|s_Roseburia_inulinivorans	-0.003±1.993	0.158±1.892	0.62	0.09	0.63
g_Anaerotruncus	0.516±1.987	-0.152±2.333	0.07	0.20	0.82
g_Anaerotruncus|s_Anaerotruncus_colihominis	0.253±1.595	-0.067±1.894	0.28	0.43	0.84
g_Acidaminococcus	0.052±1.385	-0.321±1.421	0.11	0.15	0.77
g_Acidaminococcus|s_Acidaminococcus_unclassified	0.046±1.611	-0.184±1.239	0.34	0.36	0.84
***TRPM7 genotype: GA* (n=86)**					
	**Rectal Mucosa**				
g_Eggerthella	-0.114±0.728	0.096±0.623	0.16	0.30	0.84
g_Eggerthella|s_Eggerthella_unclassified	-0.113±0.723	0.096±0.623	0.16	0.30	0.84
g_Bacteroides|s_Bacteroides_dorei	0.370±2.623	-0.278±2.137	0.22	0.33	0.84
g_Bacteroides|s_Bacteroides_faecis	0.005±1.040	0.014±1.471	0.97	0.94	0.95
g_Barnesiella	0.218±1.844	0.639±2.706	0.41	0.20	0.82
g_Barnesiella|s_Barnesiella_intestinihominis	0.218±1.844	0.639±2.706	0.41	0.20	0.82
g_Parabacteroides|s_Parabacteroides_unclassified	0.024±2.252	0.801±1.855	0.09	0.36	0.84
g_Prevotella|s_Prevotella_copri	0.335±2.219	-0.005±2.767	0.54	0.85	0.95
g_Clostridium	0.014±1.713	0.277±1.872	0.51	0.87	0.95
g_Clostridium|s_Clostridium_symbiosum	0.144±1.361	0.188±0.850	0.86	0.64	0.90
g_Flavonifractor	0.128±1.139	0.198±0.901	0.76	0.88	0.95
g_Flavonifractor|s_Flavonifractor_plautii	0.128±1.139	0.198±0.901	0.76	0.88	0.95
g_Blautia	0.263±3.110	0.406±2.557	0.82	0.81	0.95
g_Blautia|s_Ruminococcus_torques	0.149±3.019	0.294±2.465	0.81	0.82	0.95
g_Lachnospiraceae_noname|s_Lachnospiraceae_bacterium_3_1_57FAA_CT1	-0.402±1.681	0.199±0.925	0.05	0.91	0.95
g_Roseburia	-0.056±2.715	0.020±2.425	0.89	0.36	0.84
g_Roseburia|s_Roseburia_intestinalis	0.180±1.659	0.015±1.855	0.67	0.93	0.95
g_Roseburia|s_Roseburia_inulinivorans	0.119±1.479	0.524±1.471	0.22	0.52	0.84
g_Anaerotruncus	0.022±1.066	0.000±0.000	0.89	0.23	0.83
g_Anaerotruncus|s_Anaerotruncus_colihominis	0.022±1.066	0.000±0.000	0.89	0.23	0.83
g_Acidaminococcus	0.098±1.295	0.350±2.022	0.50	0.35	0.84

g_Acidaminococcus|s_Acidaminococcus_unclassified	0.099±1.294	0.349±2.019	0.51	0.36	0.84
**Rectal Swab**					
g_Eggerthella	0.025±2.084	0.656±2.046	0.16	0.31	0.84
g_Eggerthella|s_Eggerthella_unclassified	0.023±2.084	0.589±2.017	0.20	0.39	0.84
g_Bacteroides|s_Bacteroides_dorei	-0.072±1.246	-0.332±1.144	0.32	0.27	0.84
g_Bacteroides|s_Bacteroides_faecis	0.045±1.426	0.071±1.298	0.93	0.74	0.93
g_Barnesiella	0.029±0.691	0.129±1.512	0.69	0.66	0.90
g_Barnesiella|s_Barnesiella_intestinihominis	0.029±0.691	0.129±1.512	0.69	0.66	0.90
g_Parabacteroides|s_Parabacteroides_unclassified	-0.122±2.337	0.411±1.402	0.20	0.35	0.84
g_Prevotella|s_Prevotella_copri	-0.263±1.217	0.599±1.233	0.008**	0.007**	0.39
g_Clostridium	-0.524±1.440	0.275±1.984	0.04*	0.03*	0.45
g_Clostridium|s_Clostridium_symbiosum	-0.360±1.634	0.255±1.715	0.09	0.04*	0.45
g_Flavonifractor	0.096±2.517	0.113±1.917	0.97	0.53	0.84
g_Flavonifractor|s_Flavonifractor_plautii	0.096±2.517	0.113±1.917	0.97	0.53	0.84
g_Blautia	-0.036±2.328	0.042±1.176	0.84	0.25	0.84
g_Blautia|s_Ruminococcus_torques	-0.306±2.380	0.237±1.784	0.23	0.05	0.45
g_Lachnospiraceae_noname|s_Lachnospiraceae_bacterium_3_1_57FAA_CT1	-0.823±1.983	0.268±2.327	0.02*	0.05	0.45
g_Roseburia	0.326±1.782	-0.195±1.371	0.13	0.74	0.93
g_Roseburia|s_Roseburia_intestinalis	0.017±2.923	-0.211±2.357	0.69	0.96	0.96
g_Roseburia|s_Roseburia_inulinivorans	-0.054±1.880	0.010±1.978	0.88	0.75	0.93
g_Anaerotruncus	0.472±2.294	0.201±2.203	0.58	0.75	0.93
g_Anaerotruncus|s_Anaerotruncus_colihominis	0.194±2.181	0.323±1.899	0.77	0.96	0.96
g_Acidaminococcus	-0.463±1.205	0.158±1.376	0.03*	0.005**	0.39
g_Acidaminococcus|s_Acidaminococcus_unclassified	-0.369±1.119	0.275±1.620	0.03*	0.01*	0.39
**Stool**					
g_Eggerthella	0.379±1.283	0.203±1.543	0.57	0.29	0.84
g_Eggerthella|s_Eggerthella_unclassified	0.379±1.279	0.169±1.484	0.48	0.21	0.83
g_Bacteroides|s_Bacteroides_dorei	-0.241±0.735	-0.209±1.282	0.89	0.79	0.95
g_Bacteroides|s_Bacteroides_faecis	-0.134±0.797	0.353±1.121	0.02*	0.03*	0.45
g_Barnesiella	-0.217±1.001	0.218±0.995	0.05	0.05	0.45
g_Barnesiella|s_Barnesiella_intestinihominis	-0.217±1.001	0.218±0.995	0.05	0.05	0.45
g_Parabacteroides|s_Parabacteroides_unclassified	0.167±1.723	0.294±1.933	0.75	0.80	0.95

g_Prevotella|s_Prevotella_copri	-0.234±1.295	0.386±1.295	0.03*	0.04*	0.45
g_Clostridium	-0.094±0.858	0.142±1.459	0.37	0.68	0.90
g_Clostridium|s_Clostridium_symbiosum	-0.195±1.161	0.066±1.500	0.37	0.37	0.84
g_Flavonifractor	-0.240±1.515	0.100±1.769	0.34	0.44	0.84
g_Flavonifractor|s_Flavonifractor_plautii	-0.240±1.515	0.100±1.769	0.34	0.44	0.84
g_Blautia	0.263±1.085	0.060±1.218	0.42	0.12	0.76
g_Blautia|s_Ruminococcus_torques	0.048±1.653	0.206±1.677	0.66	0.68	0.90
g_Lachnospiraceae_noname|s_Lachnospiraceae_bacterium_3_1_57FAA_CT1	-0.466±1.323	0.133±1.590	0.06	0.08	0.63
g_Roseburia	0.014±1.039	-0.115±0.905	0.54	0.65	0.90
g_Roseburia|s_Roseburia_intestinalis	-0.164±1.816	0.049±1.885	0.59	0.79	0.95
g_Roseburia|s_Roseburia_inulinivorans	-0.268±1.541	0.066±2.039	0.40	0.50	0.84
g_Anaerotruncus	0.180±2.047	0.326±2.080	0.74	0.80	0.95
g_Anaerotruncus|s_Anaerotruncus_colihominis	0.212±1.453	0.145±1.633	0.84	0.67	0.90
g_Acidaminococcus	-0.125±1.108	0.020±1.162	0.56	0.23	0.83
g_Acidaminococcus|s_Acidaminococcus_unclassified	-0.052±1.002	0.007±1.149	0.80	0.43	0.84

Mean ± standard deviation.
Generalized linear model were used: *P*1 not adjusted; *P*2 adjusted for age, sex, BMI and baseline level.
*p<0.05

Supplementary Figures

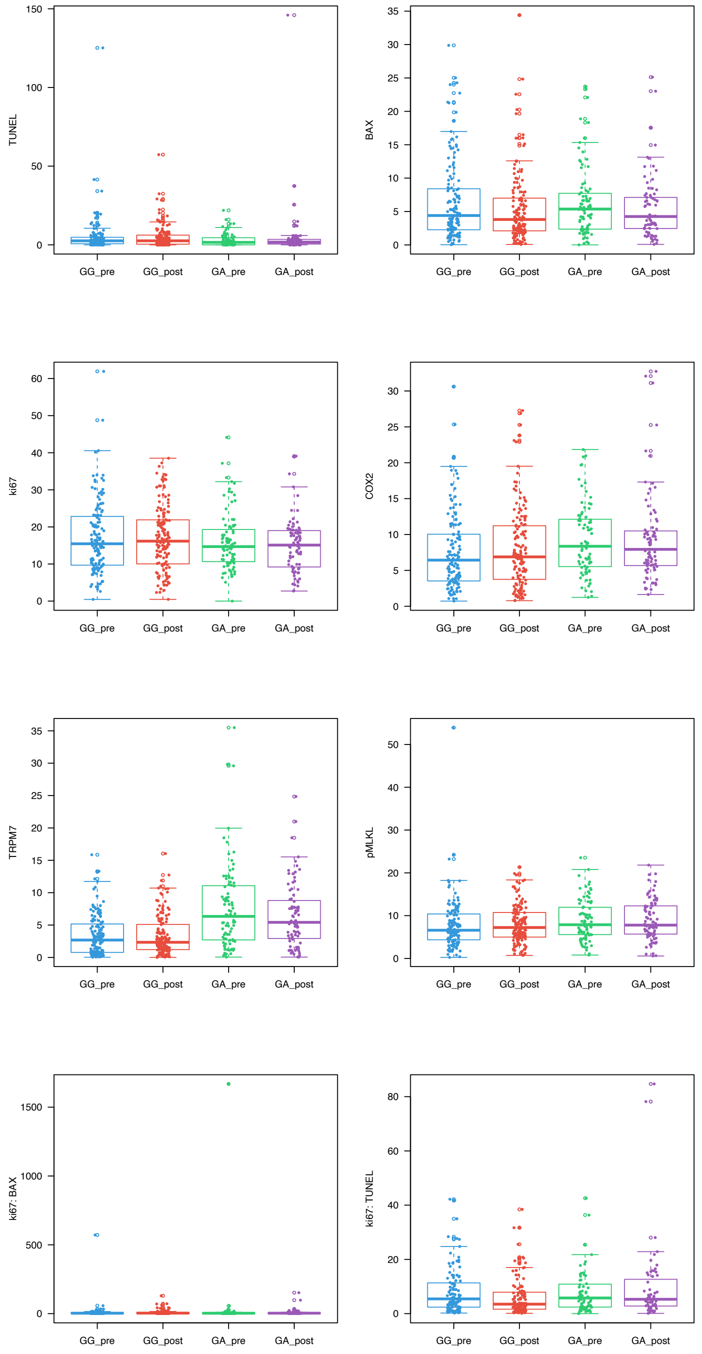

Supplementary Figure 1. IHC markers at the baseline and post-treatment time points of participants with GG and GA genotypes.

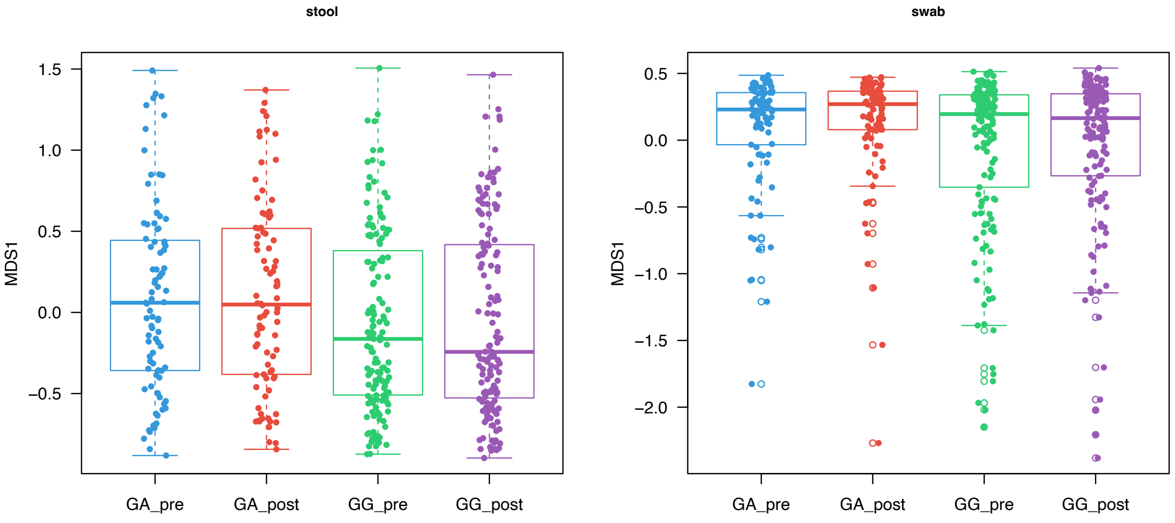

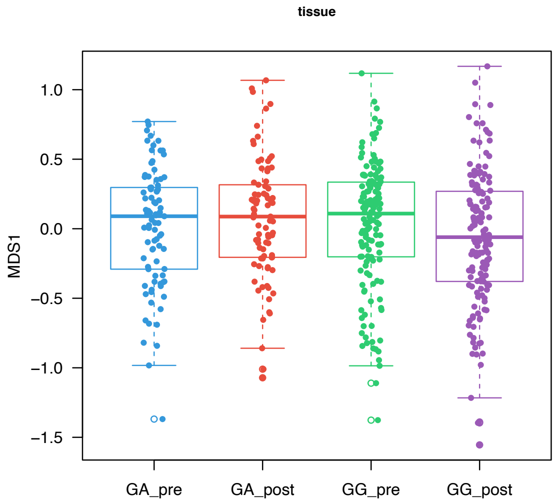

Group	R2	P
stool_post	0.00618	0.111
stool_pre	0.00589	0.153
swab_post	0.00467	0.323
swab_pre	0.00528	0.241
tissue_post	0.00738	0.078
tissue_pre	0.00690	0.104

Supplementary Figure 2. Boxplots of MDS1 by TRPM7 genotypes (GA and GG) and timepoints (pre and post-treatment) in stool, rectal swab and rectal mucosa tissue samples and PERMANOVA tests of microbial abundance and genotype in each combination of sample type and timepoints.

Stool

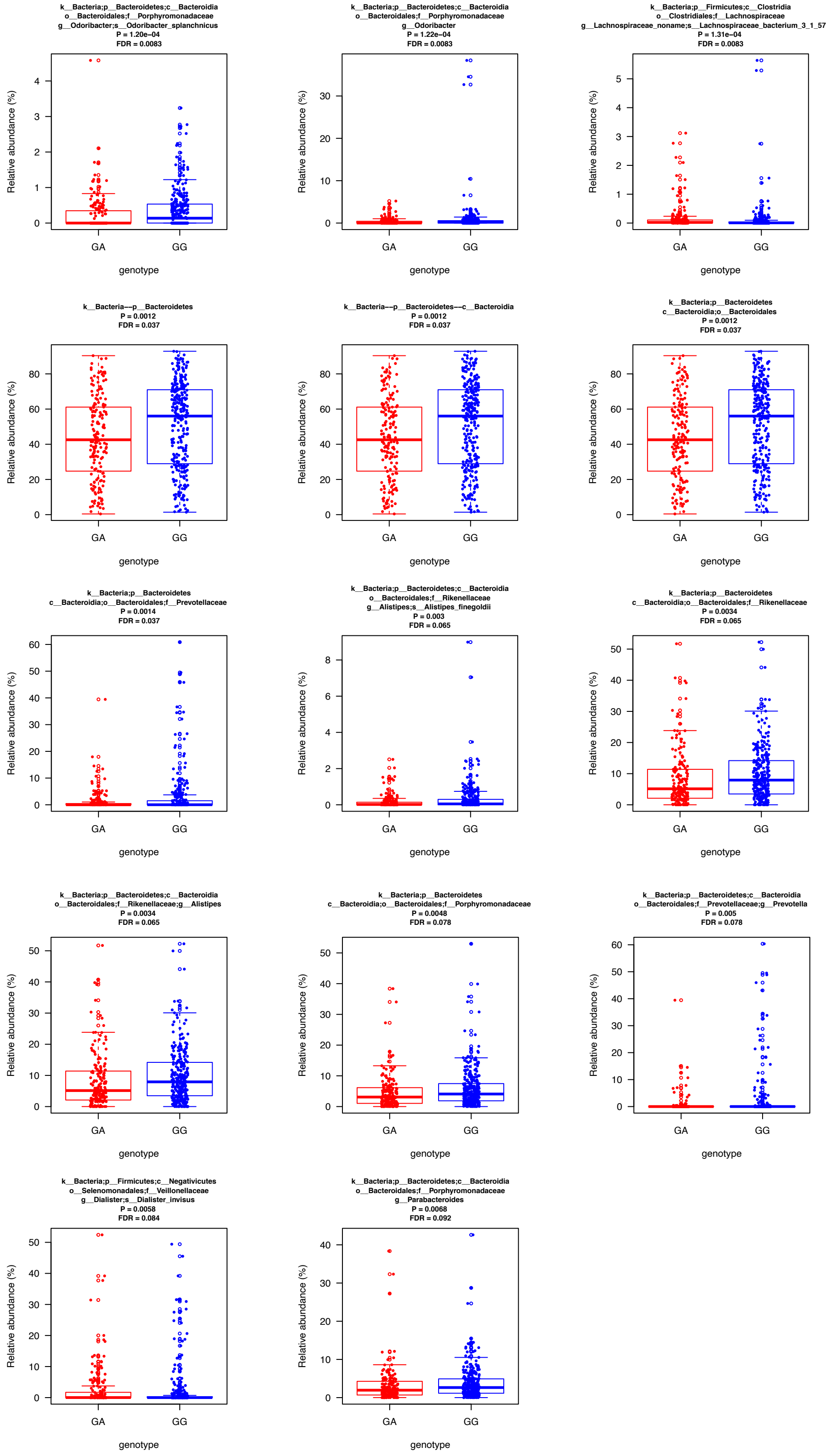
k__Bacteria;p__Bacteroidetes;c__Bacteroidia
o__Bacteroidales;f__Porphyromonadaceae
g__Odoribacter;s__Odoribacter_splanchnicus
P = 1.20e–04
FDR = 0.0083
k__Bacteria;p__Bacteroidetes;c__Bacteroidia
o__Bacteroidales;f__Porphyromonadaceae
g__Odoribacter
P = 1.22e–04
FDR = 0.0083
k__Bacteria;p__Firmicutes;c__Clostridia
o__Clostridiales;f__Lachnospiraceae
g__Lachnospiraceae_noname;s__Lachnospiraceae_bacterium_3_1_57
P = 1.31e–04
FDR = 0.0083
k__Bacteria––p__Bacteroidetes
P = 0.0012
FDR = 0.037
k__Bacteria––p__Bacteroidetes––c__Bacteroidia
P = 0.0012
FDR = 0.037
k__Bacteria;p__Bacteroidetes
c__Bacteroidia;o__Bacteroidales
P = 0.0012
FDR = 0.037
k__Bacteria;p__Bacteroidetes
c__Bacteroidia;o__Bacteroidales;f__Prevotellaceae
P = 0.0014
FDR = 0.037
k__Bacteria;p__Bacteroidetes;c__Bacteroidia
o__Bacteroidales;f__Rikenellaceae
g__Alistipes;s__Alistipes_finegoldii
P = 0.003
FDR = 0.065
k__Bacteria;p__Bacteroidetes
c__Bacteroidia;o__Bacteroidales;f__Rikenellaceae
P = 0.0034
FDR = 0.065
k__Bacteria;p__Bacteroidetes;c__Bacteroidia
o__Bacteroidales;f__Rikenellaceae;g__Alistipes
P = 0.0034
FDR = 0.065
k__Bacteria;p__Bacteroidetes
c__Bacteroidia;o__Bacteroidales;f__Porphyromonadaceae
P = 0.0048
FDR = 0.078
k__Bacteria;p__Bacteroidetes;c__Bacteroidia
o__Bacteroidales;f__Prevotellaceae;g__Prevotella
P = 0.005
FDR = 0.078
k__Bacteria;p__Firmicutes;c__Negativicutes
o__Selenomonadales;f__Veillonellaceae
g__Dialister;s__Dialister_invisus
P = 0.0058
FDR = 0.084
k__Bacteria;p__Bacteroidetes;c__Bacteroidia
o__Bacteroidales;f__Porphyromonadaceae
g__Parabacteroides
P = 0.0068
FDR = 0.092

Swab

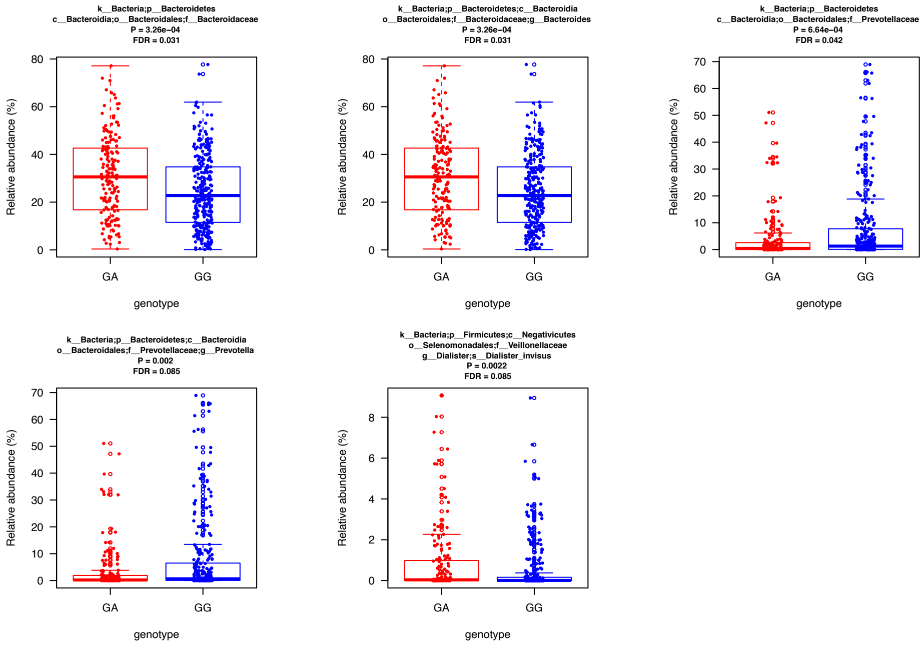

Supplementary Figure 3. Boxplots of taxonomic compositions (at taxonomic levels of phylum, class, order, family, genus, species, strains) that are significantly different between genotypes in stool, rectal swab or rectal mucosa tissue samples (Wilcoxon test, FDR<0.1). There were no significantly different taxa in mucosa tissue samples.

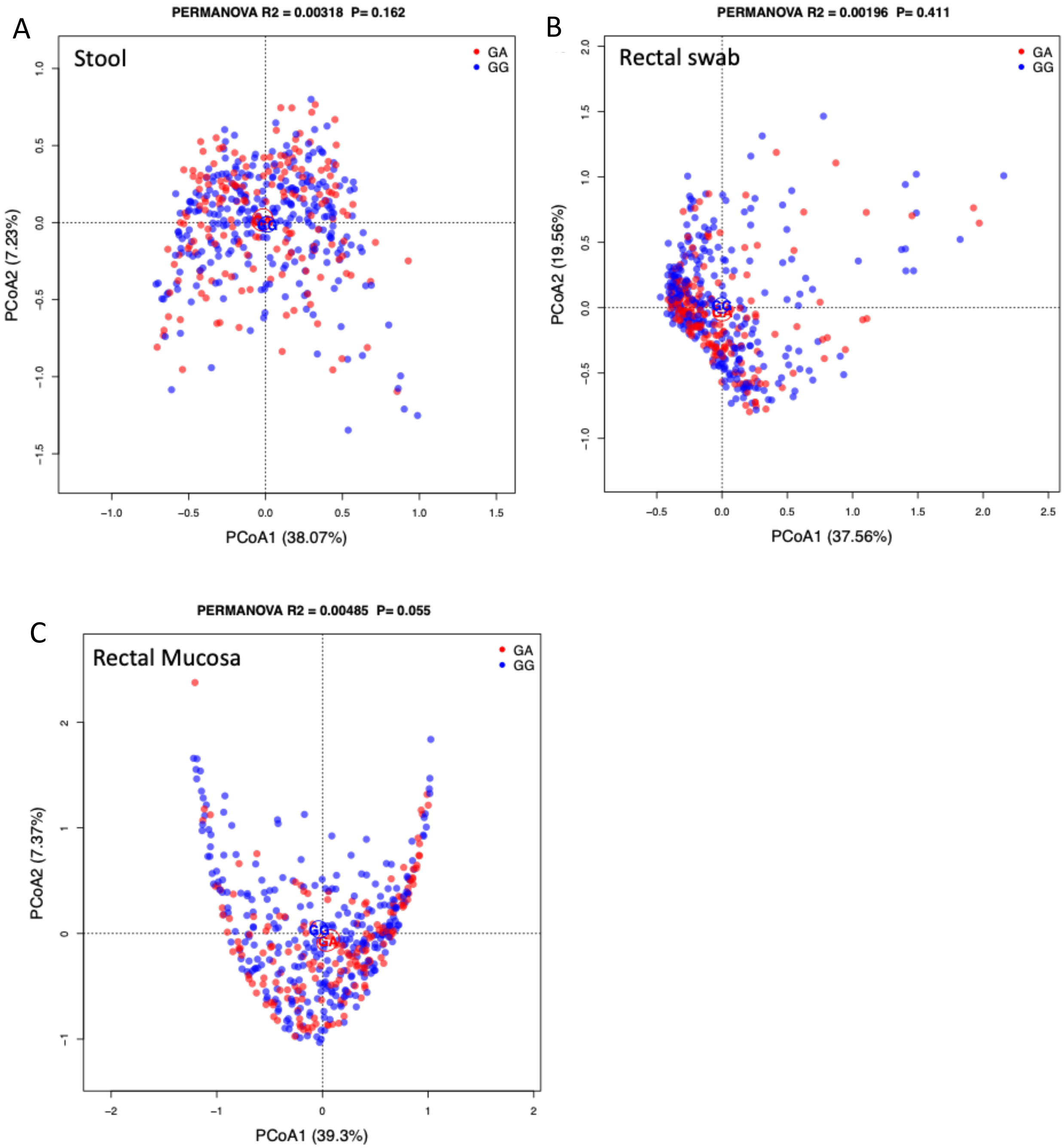

Supplementary Figure 4. Multidimensional scaling of unstratified functional pathway profiles of stool, swab and tissue samples of study subjects. Stool (A), swab (B) and tissue (C) samples were colored based on genotype.

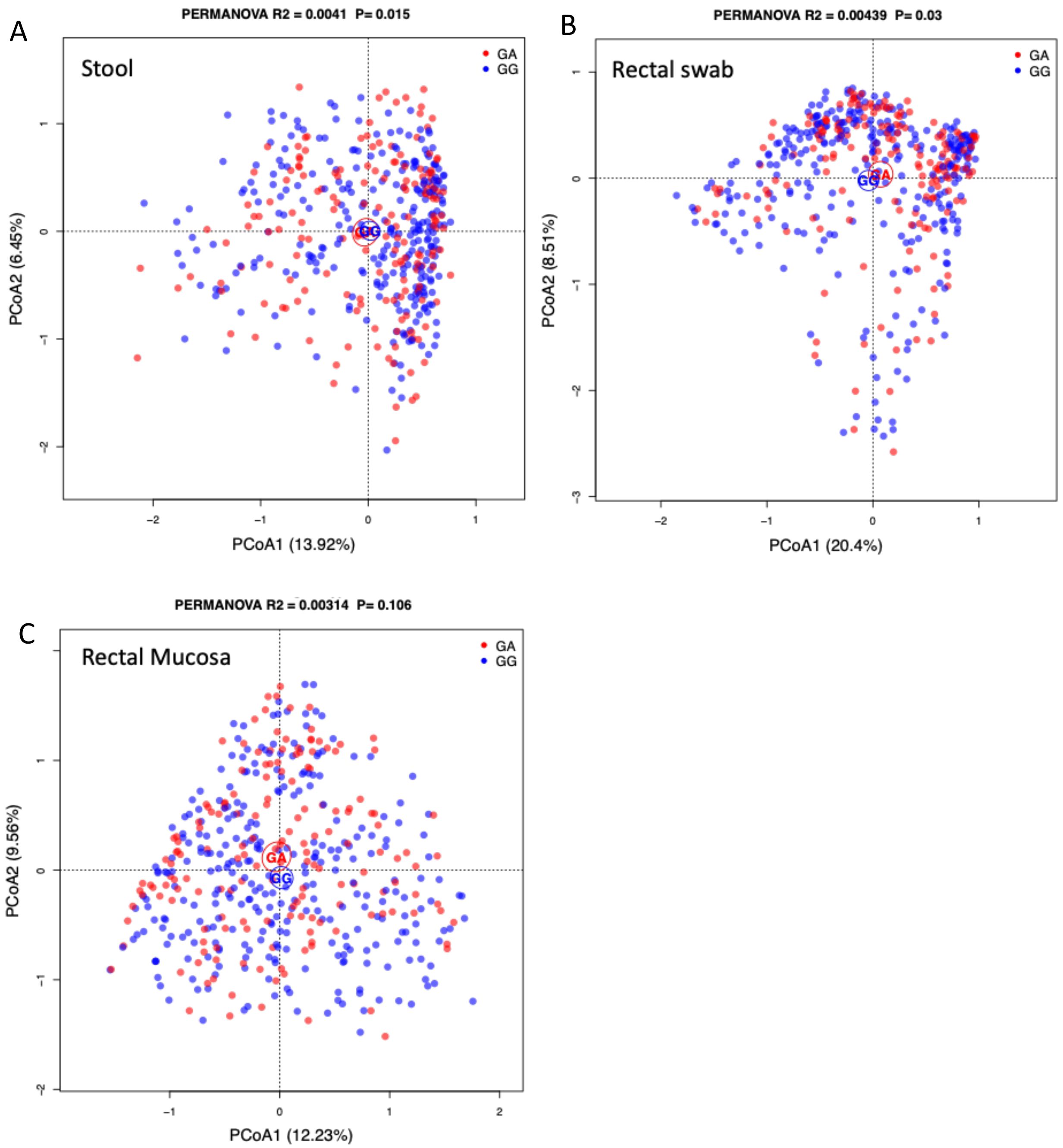

Supplementary Figure 5. Multidimensional scaling of taxa-stratified functional pathway profiles of stool, swab and tissue samples of study subjects. Stool (A), swab (B) and tissue (C) samples were colored based on genotype.

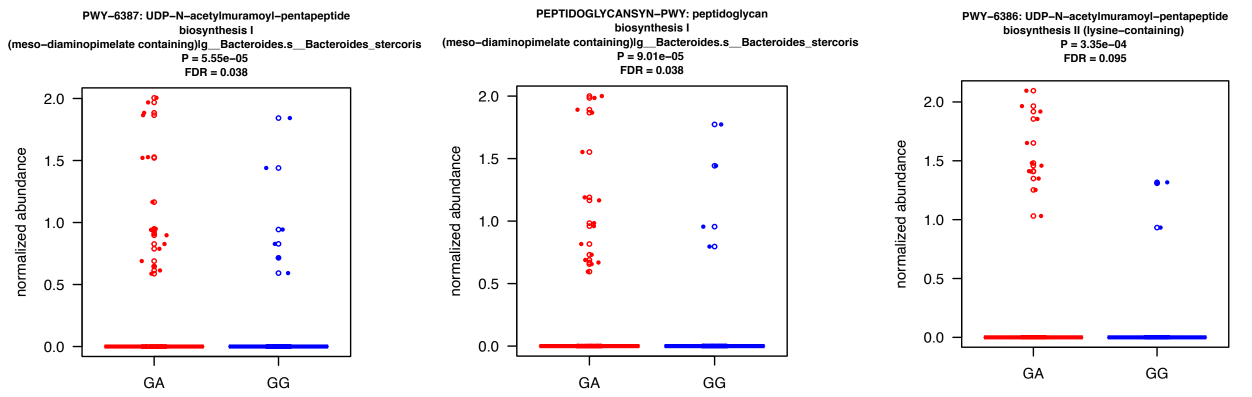

Supplementary Figure 6. Boxplots of functional pathway profiles that are significantly different between genotypes in tissue samples (Wilcoxon test, FDR<0.1), including both taxa-stratified and unstratified pathway profiles.

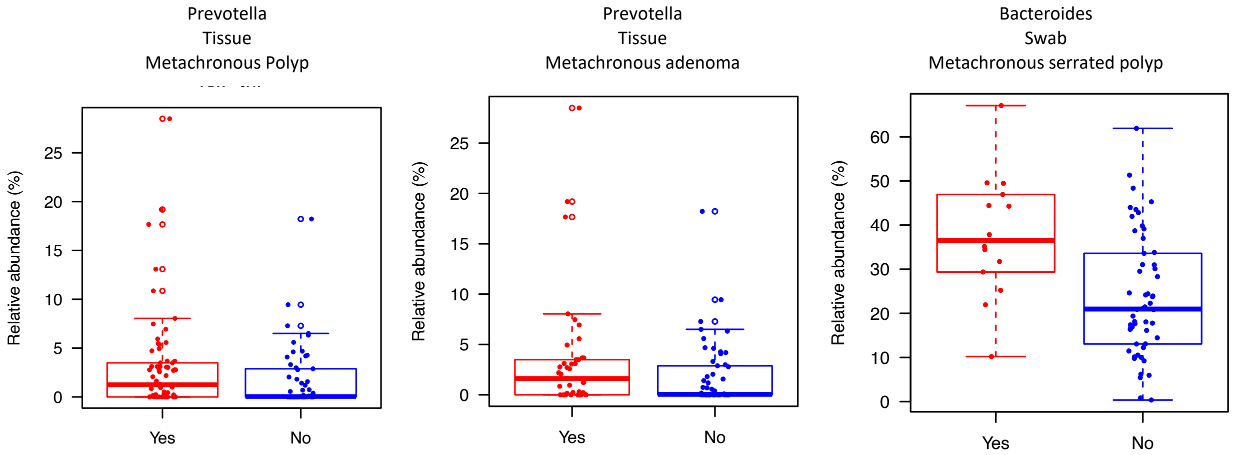

Supplementary Figure 7. Significant associations between the post-trial abundance of *Prevotella*, *Bacteroides* and metachronous colorectal polyps (test results are shown in Table 3).

Stool
Metachronous polyp (adenoma/serrated polyp)

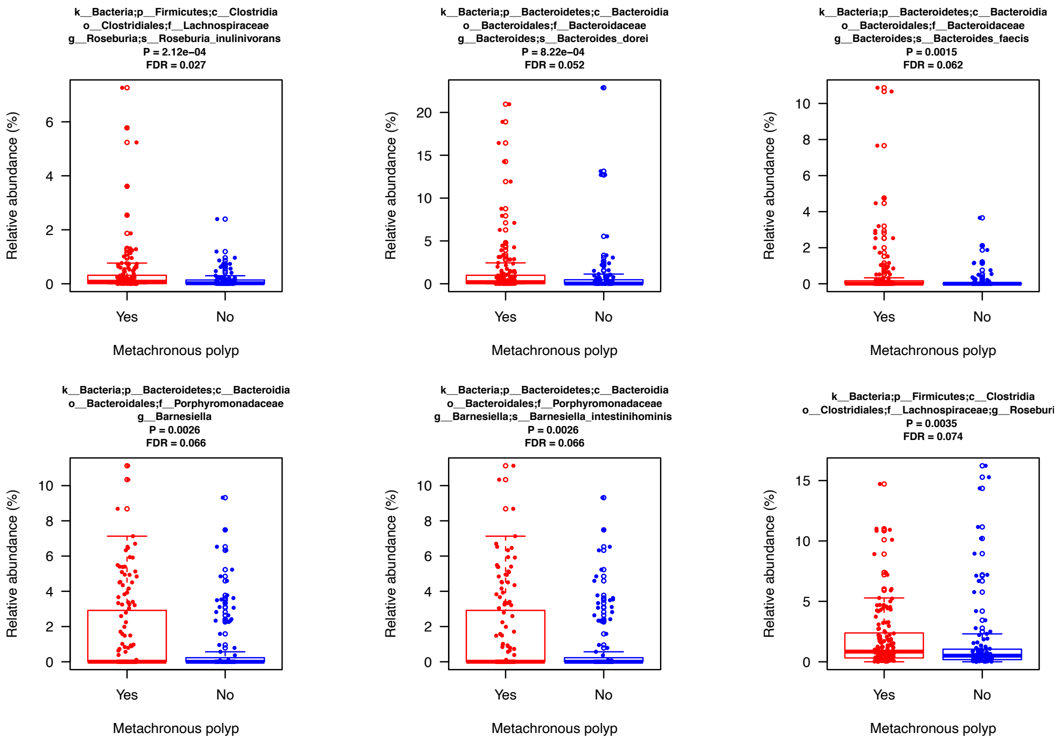

Stool

Metachronous adenoma

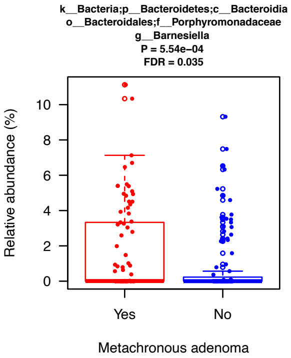

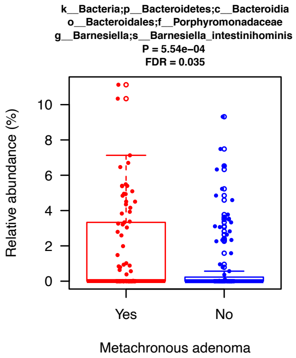

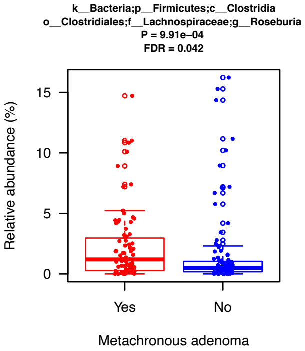

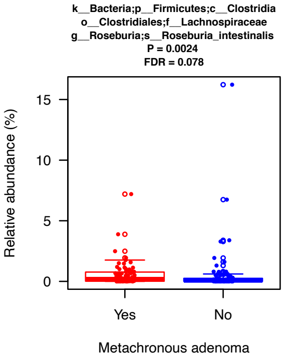

Metachronous serrated polyp

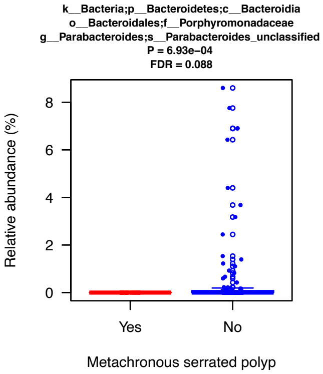

Swab

Metachronous polyp (adenoma/serrated polyp)

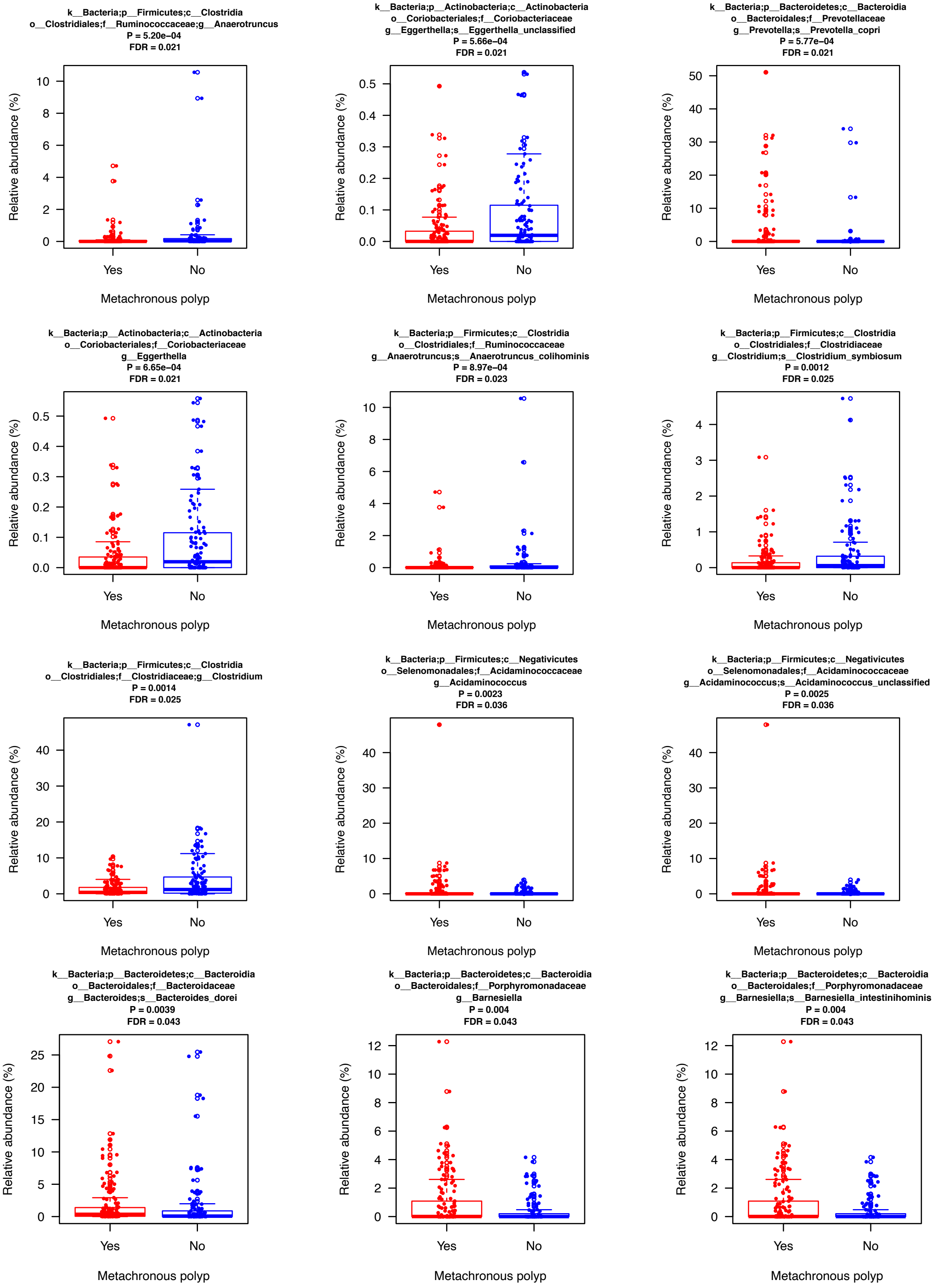

Swab
Metachronous adenoma

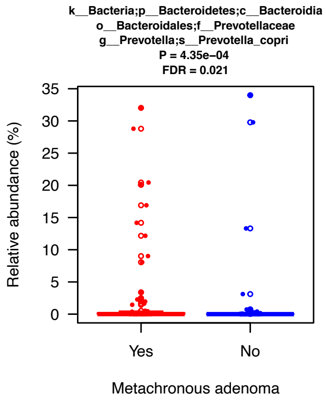

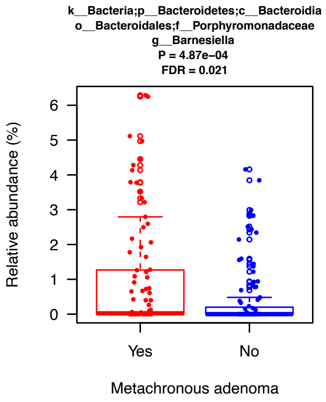

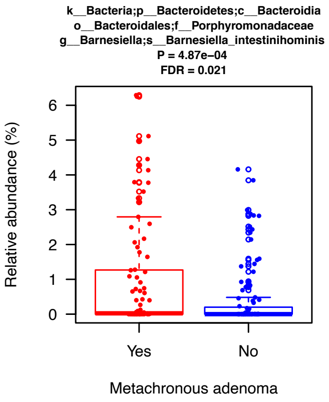

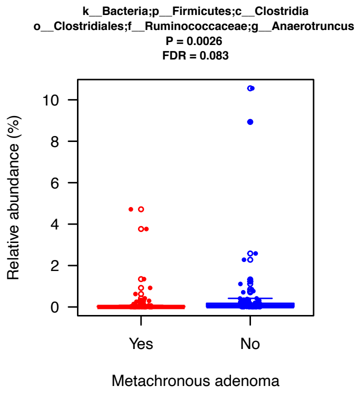

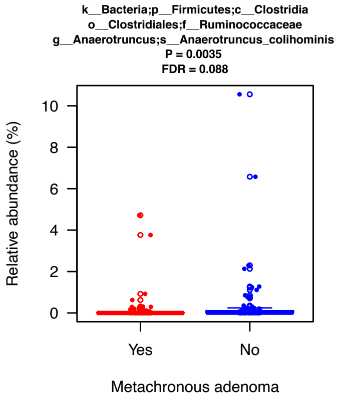

Swab

Metachronous serrated polyp

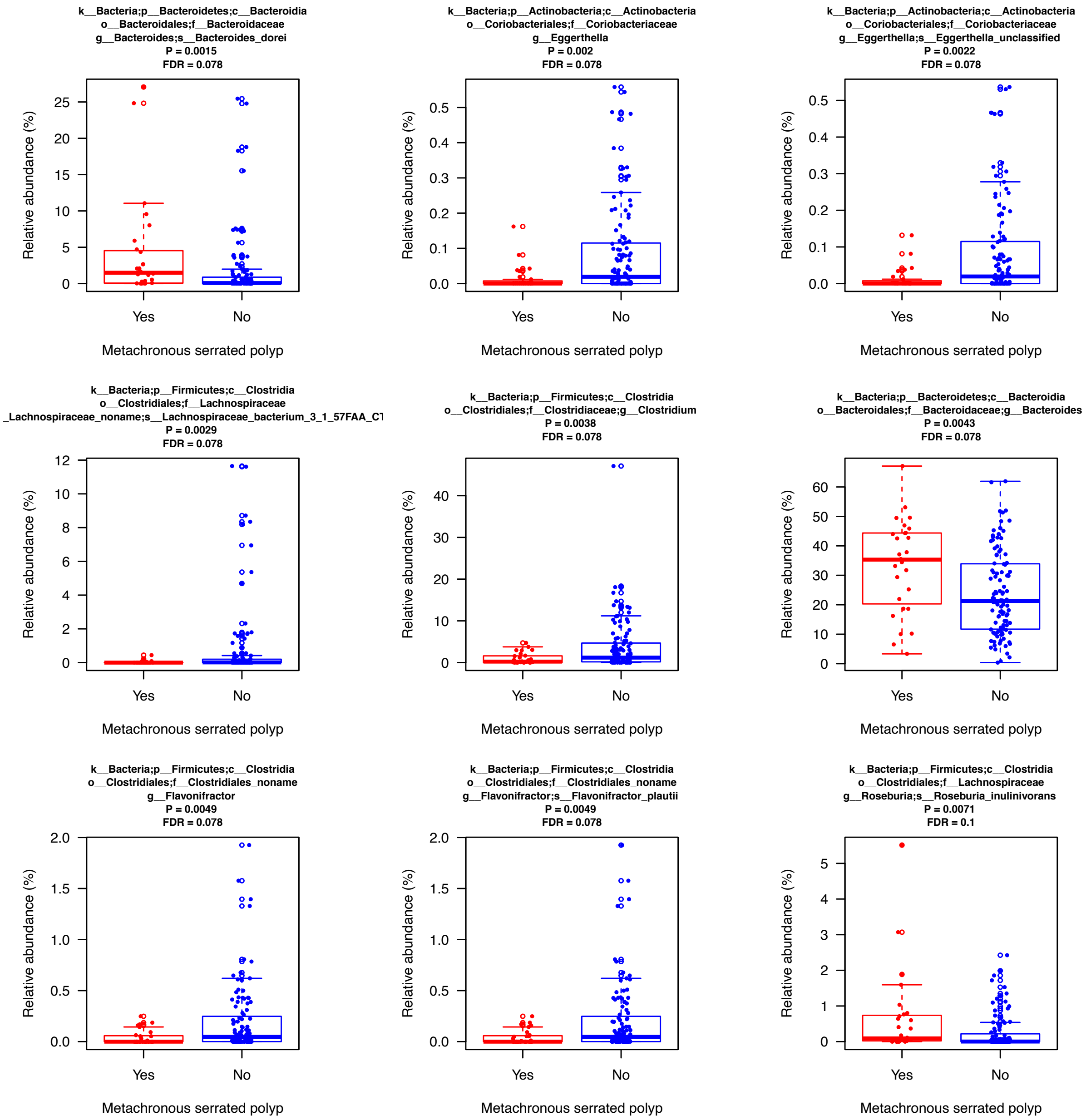

Tissue

Metachronous polyp (adenoma/serrated polyp)

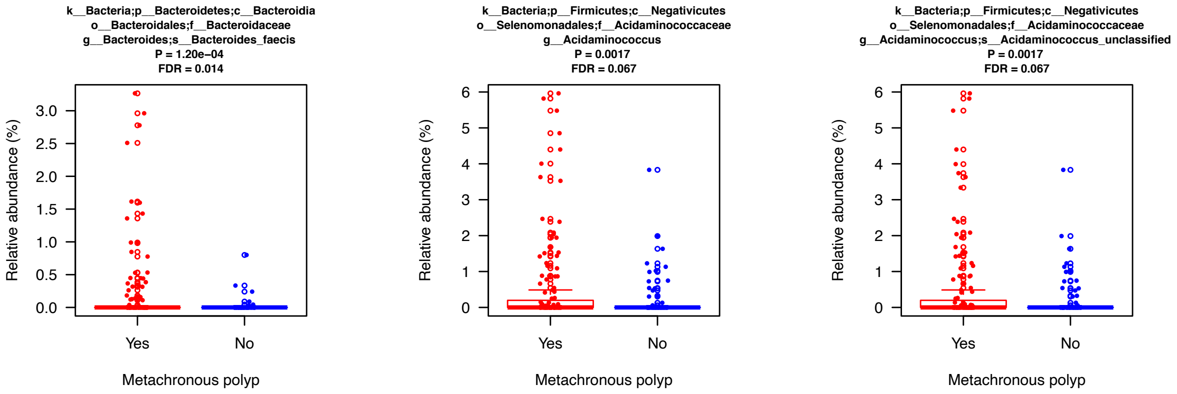

Tissue

Metachronous adenoma

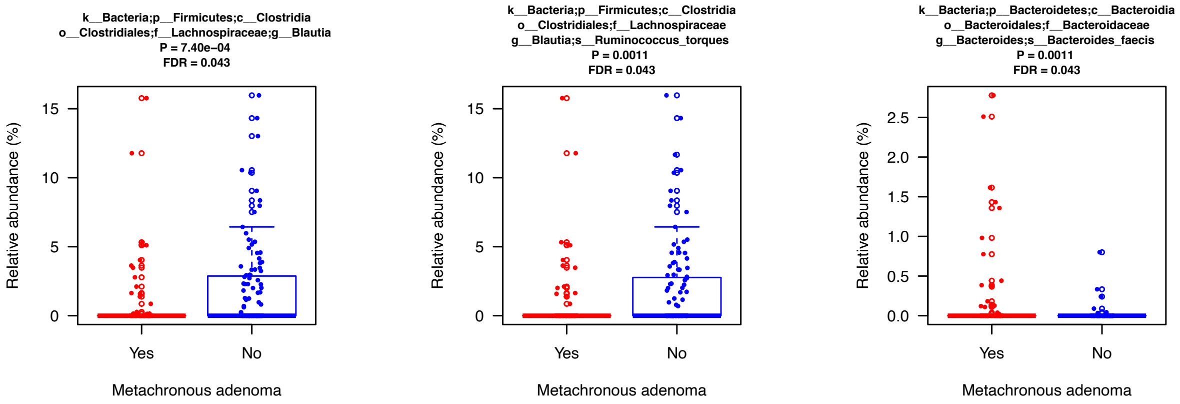

Supplementary Figure 8. Significant associations between taxa (phylum to species level) and metachronous colorectal polyps, adenoma and serrated polyps (Wilcoxon, FDR<0.1) in stool, rectal swab and rectal mucosa tissue. There were no significant associations with serrated polyps in stool and tissue samples. Samples at the beginning and the end of the trial were pooled for this analysis.

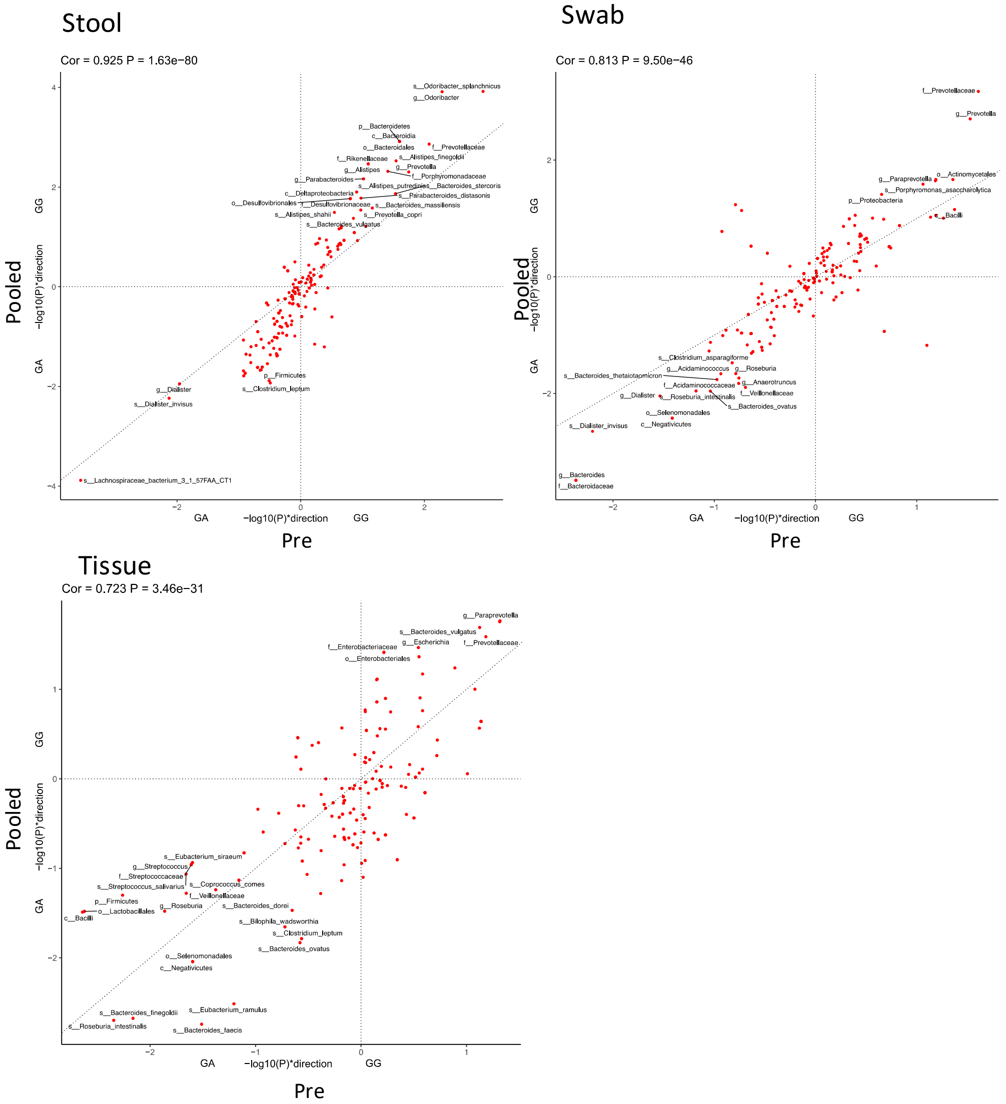

Supplementary Figure 9. Comparison of genotype-associated taxa in samples collected at the beginning of the trial and taxa in samples collected at both the beginning and the end of trial. The x-axis is the -log10 P-value from Wilcoxon test comparing GA and GG genotypes in pre timepoints, multiplied by the direction of changes. The y-axis is the same -log10 P-value multiplied by the direction of changes in pooled pre and post timepoints. The GA and GG on the axes indicate which genotype the microbial taxa were enriched in.
